# Protocol for real-time monitoring of CD8^+^ T and myeloid cell behavior in human high-grade serous ovarian cancer slices

**DOI:** 10.1016/j.xpro.2024.103102

**Published:** 2024-06-02

**Authors:** Florian Laforêts, Emmanuel Donnadieu, Frances Balkwill

**Affiliations:** 1 Barts Cancer Institute, Queen Mary University of London, Charterhouse Square, EC1M6BQ London, UK; 2 Université Paris Cité, CNRS, INSERM, Equipe Labellisée Ligue Contre le Cancer, Institut Cochin, 75014 Paris, France

**Keywords:** Cancer, Immunology, Microscopy

## Abstract

Studying cell behavior in live human tumors is crucial to understand and improve response to immunotherapies. Here, we present a protocol to slice human ovarian tumors *ex vivo*, maintain their viability for 24 h, and monitor the behavior of CD8^+^ T and myeloid cells in real time. Furthermore, we detail procedures to semi-automatically analyze cell movements and aggregate and process behavior data. This protocol can potentially be applied for multiple tumor types and mouse cancer models.

For complete details on the use and execution of this protocol, please refer to Laforets et al.^1^

## Highlights

• Human high-grade serous ovarian tumor slices are kept viable up to 24 h *ex vivo*
• Steps to monitor CD8+ T and myeloid cell movement in real time in *ex vivo* tumor slices
• Instructions for tracking and quantifying movement of CD8^+^ T and myeloid cells
• Movement data aggregation and behavioral analysis of both cell types are automated

## Abstract (editor highlights)

Publisher’s note: Undertaking any experimental protocol requires adherence to local institutional guidelines for laboratory safety and ethics.

## Graphical abstract

**Figure:**
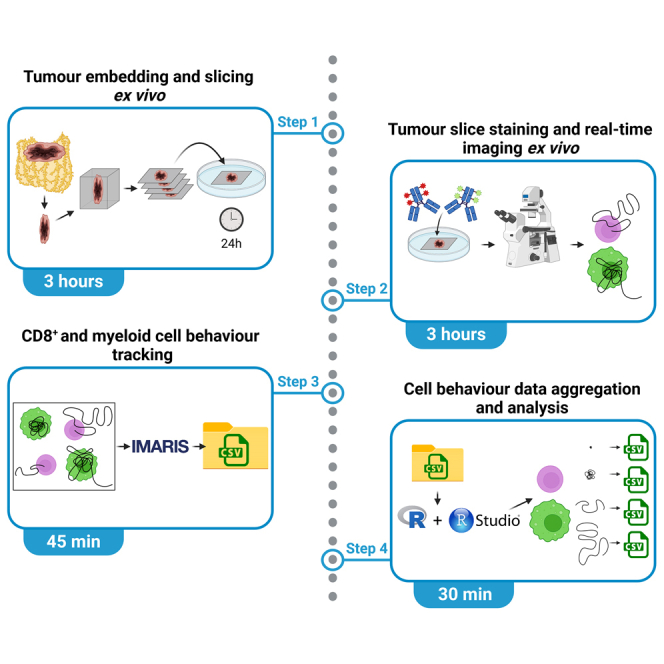

## Before you begin

This protocol describes specific steps for analyzing the behavior of CD8^+^ T and myeloid cells in human *ex vivo* slices of high-grade serous ovarian cancer omental biopsies, shortly after resection and up to 24 h after slicing, providing the opportunity for treatment of the slices. However, we have also used this protocol to study mouse models of ovarian cancer, and we believe it can be used to study other cancers and 3D models. In addition, the technique we report to categorize cell behavior could be applied to other cell types and biological settings.

### Institutional permissions

#### Patient samples

Human high grade serous ovarian cancer specimens used in this protocol were surplus to diagnosis and donated by patients, after providing written informed consent and were processed under studies approved by a UK national review board (REC 10/H0304/14, 15/EE/0151 and 17/LO/0405). All tissue processing and subsequent experiments followed the declaration of Helsinki and the international ethical guidelines for biomedical research involving human subjects.

The use of human specimens in this protocol requires obtaining written consent from patients and permission from the relevant institutions, observing national guidelines and regulation.

### Preparing the *ex vivo* tumor slice media

**Timing: 1 h**

The details in this section are for one 500 mL bottle of media.

1. Preparing stock solutions for supplements. All these can be prepared at room temperature.
  a. Open the bottle of RPMI 1640 with phenol red and set aside 58 mL in two 50 mL tubes.
  b. Prepare 50 μg/mL hydrocortisone stock solution. Once prepared, this solution can be stored at 4°C. We have used it up to one month after reconstitution.
    i. Re-suspend hydrocortisone with 1 mL of 100% ethanol.
    ii. Add 19 mL of RPMI 1640 with phenol red (from the set aside media).
  c. Prepare 0.5 mg/mL recombinant human EGF stock solution.
    i. Briefly centrifuge the vial containing the EGF before reconstitution to bring content at the bottom.
    ii. Reconstitute in 1 mL of sterile ddH_2_O. This can be aliquoted and kept at −20°C.
      ***Note:*** Once reconstituted we recommend aliquoting and storing at −20°C. We have used aliquots up to 6 months after reconstitution.
  d. Prepare 1 mg/mL fetuin by dissolving 10 mg in 10 mL sterile ddH_2_O. This solution can be stored at 4°C. We have used it up to one month after reconstitution.
  e. Prepare 20 μg/mL 3,3′,5-Triiodo-L-thyronine (TIT) stock solution. This solution can be stored at 4°C. We have used it up to one month after reconstitution.
    i. Dissolve 1 mg TIT in 1 mL 1 M NaOH.
    ii. Add 49 mL of RMPI 1640 with phenol red (from the media set aside).
  f. Prepare 0.5 M O-Phosphorylethanolamine by dissolving 70.53 mg in 1 mL sterile ddH_2_O. This solution can be stored −20°C. We have used it up to 6 months after reconstitution.
2. Supplement the RPMI 1640 (with phenol red) media. Once supplemented, the media can be used up once month and must be stored at 4°C.
  a. Add 25 mL human serum.
  b. Add 5 mL Antibiotic-Antimycotic (anti-anti) 100X.
  c. Add 5 mL Penicillin-Streptomycin (Pen-Strep) 100X.
  d. Add 10 mL Insulin-Transferrin-Selenium-Sodium Pyruvate (ITS-A) 100X (final 2X).
  e. Add 5 mL hydrocortisone 50 μg/mL.
  f. Add 5 mL MEM vitamins 100X.
  g. Add 3.125 mL Fetuin 1 mg/mL.
  h. Add 10 μL of recombinant human EGF 0.5 mg/mL.
  i. Dissolve 0.5 *g* BSA for cell culture in the medium.
  j. Dilute 20 μg/mL TIT 1:10 (2 μg/mL). Add 8.41 μL of the 2 μg/mL solution to the media.
  k. Add 5 μL O-Phosphorylethanolamine 0.5M.
  l. Dilute Ethanolamine 98% 1:100 in RPMI 1640 with phenol red and add 15 μL of the diluted solution to the media.
  m. Mix the media by gently inverting the bottle. The total volume should be 500.163 mL. The media can be stored at 4°C. We have used it up to one month after preparation.

### Preparing the plate + washers

**Timing: 1 h**

This protocol uses 6-well plates containing organotypic inserts.

***Note:*** Ahead of preparing to slice the tissue, plan the number of conditions and/or wells. The number of slices that it is possible to produce from a tumor sample varies greatly with sample size and content. This can be due to the variable amount of tumor material obtained from surgery as surplus to diagnosis, but also to the adipose or necrotic content. For example, a small tumor sample (i.e., 10 × 3 × 3 mm) will yield a small number of slices. In addition, specimens that contains large amounts of adipose tissue or specimens that are visibly necrotic will need to be trimmed, thus potentially further reducing the size of the sliceable tumor specimen. However, to ensure having enough wells to place slices in, it is advisable to plan for a high number of slices and then scale down the number of conditions or slices per condition, according to the number of slices obtained.

3. Prepare 6-wells plates with 1.1 mL *ex vivo* tumor slice media per well.

***Optional:*** Add treatments to the relevant wells and mix by gently moving the plate in a forward-backward motion and in a lateral motion as well. For example, we have previously treated the slices with lipopolysaccharide.

4. Add one 30 mm organotypic insert with pore size 0.4 μm in each well. Lower the insert in the well slowly, making sure the whole surface of the PTFE membrane is hydrated.
5. Put the plate in a cell culture incubator at 37°C and 5% CO_2_ until use.
6. Prepare stainless steel washer (outside diameter 8 mm inner diameter 4 mm).
  a. Wash the stainless-steel washers in 70% ethanol.
  b. Rinse the washers twice in PBS.

***Note:*** the size of the washers should be adapted to the expected surface area of the tumor slices. For very small tumor samples, use smaller washers.

7. Prepare a 24-well plate with 750 μL formalin 10% per well to fix tumor slices, either immediately after slicing or after up to 24 h *ex vivo* culture.

### Tumor collection

**Timing: 90 min**

8. Prepare a 150 mm petri dish filled with ice cold sterile PBS under a tissue culture hood and put at 4°C until arrival of the fresh tumor sample.
9. Prepare a set of forceps to handle the tumor sample along with a scalpel to cut it.
10. Collect the tumor at the hospital.
  a. After resection, place the fresh omental biopsies of high grade serous ovarian cancer, judged to be surplus to diagnosis by the lead surgeon, in a 50 mL sample pot.
  b. Transport the specimen back to the laboratory in a cool bag containing an ice pack.

***Note:*** The sample pot must then be filled with enough ice-cold PBS to cover the specimen. In addition, the time between tumor resection and arrival the laboratory takes 90 min to 4 hours In our hands, this has not affected the tumor slice *ex vivo*.

11. Upon arrival, transfer the tumor sample to the 150 mm petri dish filled with ice cold PBS under a cell culture hood.

### Setting up the peristaltic pump and the media oxygenation

**Timing: 30 min**

**Figure 1:**
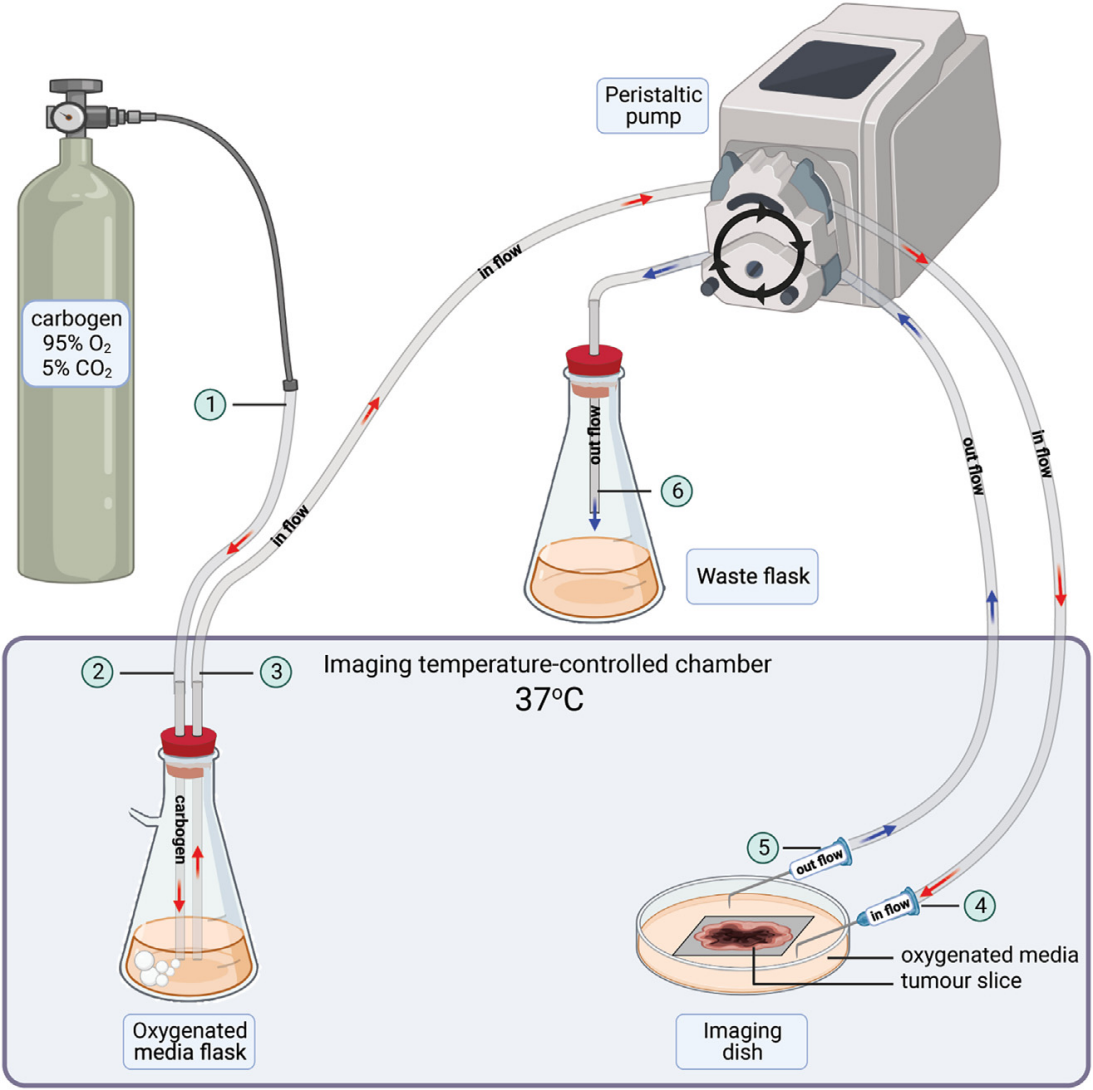
Imaging media oxygenation using carbogen and a two-channel peristaltic pump, related to before you begin step 12 A conical 500 mL flask is filled with at least 250 mL unsupplemented phenol red-free RPMI 1640 (imaging media). A carbogen cylinder equipped with a regulator is connected to a bioprene tube (end 1) plunged (end 2) in the imaging media. The pressure is set on 0.1 bar to gently bubble the medium with carbogen. The two-channel peristaltic pump is equipped with two sections of bioprene tubing. One end (3) of the “in flow” section is plunged in the bubbled imaging media while the other end (4), fitted with a bent needle, is immersed in the imaging dish. The “out flow” section begins with one end (5) fitted with a bent needle immersed in the imaging dish and ends in the waste flask (6). Both the imaging dish and the oxygenated media flask are kept inside the imaging temperature-controlled chamber, set on 37°C and 5% CO_2_.

12. Set up a peristaltic pump with two channels, each with a set of bioprene tubing (Figure 1).
  a. Set the pump outside the temperature-controlled chamber, on a different surface from the microscope to prevent the vibrations disturbing the microscope during imaging.
  b. Attach two sets of bioprene tubing to the pump, once for each channel.
  c. Set up a carbogen cylinder:
    i. Set up a regulator on 0.1 bar.
    ii. Connect the gas outlet to a bioprene tube (tube end 1).
    iii. Connect the bioprene tube end 2 to a 2 mL pipette. This will later be inserted in a stopper containing two holes, into the oxygenated media flask.
  d. Insert a 2 mL plastic pipette in the bioprene in flow tube (tube end 3). This will later be inserted in a stopper containing two holes, into the oxygenated media flask.
  e. Prepare two bent 19G 40 mm needles by bending them 6–8 mm from the tip. Wash and keep them in ethanol. These will later be attached to in flow tube end 4 and out flow tube end 5, respectively.
  f. Set up a waste conical flask (at least 1 L capacity) outside the temperature-controlled chamber.
    i. Add 100 mL Virkon.
    ii. Add a single hole stopper to the flask.
    iii. Insert a 2 mL plastic pipette in the stopper.
    iv. Insert the upper end of the pipette to the out flow section of bioprene tubing (tube end 6).

**CRITICAL:** For the peristaltic pump 120S/DM2 (Watson and Marlow) we used, we set up bioprene tubing with 1.6 mm bore and 1.6 mm wall thickness in the pump itself. These were connected (using tube connectors) to bioprene tubing with 0.8 mm bore and 1.6 mm wall thickness that fit 2 mL pipette and the 19G needles.

## Key resources table

**Table:** 

REAGENT or RESOURCE	SOURCE	IDENTIFIER
**Antibodies**		

Alexa Fluor 647 anti-human CD8 antibody Dilution 1:10	BioLegend	Cat# 344725; RRID:AB_2563451
CD326 (EpCAM) monoclonal antibody (MH99), Alexa Fluor 488, eBioscience Dilution 1:10	Thermo Fisher Scientific	Cat# 53-8326-42; RRID:AB_11219279
Alexa Fluor 594 anti-human CD11b antibody Dilution 1:10	BioLegend	Cat# 301340; RRID:AB_2563208
Fibronectin antibody (HFN7.1) (Alexa Fluor 405) Dilution 1:10	Novus Biologicals	Cat# NBP2-34633AF405; RRID: N/A
Human TruStain FcX	BioLegend	Cat# 422302 ; RRID: AB_2818986

**Biological samples**		

Human high-grade serous ovarian cancer omental metastasis samples		

**Chemicals, peptides, and recombinant proteins**		

Human serum	Merck	Cat# H4522
Penicillin-Streptomycin (Pen/Strep)	Merck	Cat# 11074440001
Insulin-Transferrin-Selenium-sodium pyruvate (ITS-A) (100X)	Gibco	Cat# 51300044
Hydrocortisone	Merck	Cat# H0135
Human EGF, AOF recombinant protein	Thermo Fisher Scientific	Cat# PHG6045
Antibiotic-Antimycotic (anti-anti)	Gibco	Cat# 15240062
O-Phosphorylethanolamine	Merck	Cat# P0503
3,3′,5-Triiodo-L-thyronine sodium salt	Merck	Cat# T6397
Fetuin from fetal bovine serum	Merck	Cat# F2379
Bovine serum albumin (BSA; low endotoxin)	Merck	Cat# A2058
RPMI 1640 medium, no phenol red	Thermo Fisher Scientific	Cat# 11835105
RPMI 1640 medium [+] L-glutamine	Gibco	Cat# 21875
10% neutral buffered formalin	Merck	Cat# HT501128-4L
MEM vitamin solution (100X) Green features	Thermo Fisher Scientific	Cat# 11120052
Ethanolamine ≥98%	Merck	Cat# E9508-100ML
DPBS (1X)	Gibco	Cat# 14190
RELY+ON VIRKON tablets	LANXESS	NA
Agarose type VII-A, low gelling temperature	Merck	Cat# A0701

**Softwares and algorithms**		

R v4.0.4	N/A	http://www.R-project.org
RStudio v1.4.1106	N/A	https://www.rstudio.com/products/rstudio/
tidyverse	N/A	https://www.tidyverse.org/
Cell Movement Data Aggregation and Processing Script	N/A	https://doi.org/10.5281/zenodo.11093978
GraphPad Prism, San Diego, CA version 9.0.0	N/A	https://www.graphpad.com
NIS-Elements	Nikon	https://www.microscope.healthcare.nikon.com/products/software/nis-elements
Illustrator 2023	Adobe	https://www.adobe.com/uk/products/illustrator.html
Bitplane Imaris 9.1 and 9.9	Oxford Instruments	https://imaris.oxinst.com/
Biorender.com	BioRender	https://www.biorender.com

**Other**		

35 mm dish | No. 1.5 coverslip | 20 mm glass diameter | uncoated	MatTek	Cat# P35G-1.5-20-C
Peel-A-Way embedding molds	Merck	Cat# E6032
Corning Costar TC-treated multiple 6-well plate	Merck	Cat# CLS3506
Corning Costar TC-treated multiple 24-well plate	Merck	Cat# CLS3527
Surgical scalpel blade No. 10	Swann-Morton	Cat# 0501
Carbogen (95% oxygen/5% carbon dioxide) cylinder	BOC	Cylinder size G (3400 L)
Derby double-edge razor blades Extra	Derby	N/A
Millicell cell culture insert, 30 mm, hydrophilic PTFE, 0.4 μm	Merck	Cat# PICM0RG50
M2.5 Stainless steel form A washers	CPC UK	Cat# FN00783
Cyanoacrylate glue 10 *g*	Leica Biosystems	14037127414
A2 Stainless steel washers (form A)/M4	UK Stainless	none
Slice anchor – flat stainless steel for RC-41 chamber, 1.5 mm, SHD-41/15	Warner Instruments	Cat# 641418
Slice anchor – flat stainless steel for RC-41 chamber, 1.5 mm, SHD-41/10	Warner Instruments	Cat# 641419
Graefe forceps	Fine Science Tools	Cat# 11050-10
Nikon Eclipse TE	Nikon	N/A
35 mm holder stage insert	Nikon	Cat# MXU91993
Peristaltic pump 120S/DM2	Watson-Marlow	Cat# 010.6131.M20
Vibratome VT1200S	Leica Biosystems	N/A
Bioprene tubing 0.8 mm bore x 1.6 mm wall thickness	Watson-Marlow	Cat# 933.0008.J16
Bioprene tubing 1.6 mm bore x 1.6 mm wall thickness	Watson-Marlow	Cat# 933.0016.J16
Straight connectors for 0.5 and 0.8 mm bore	Watson-Marlow	Cat# 999.2008.000
Straight connectors for 1.6 and 3.2 mm bore	Watson-Marlow	Cat# 999.2032.000
Aspiration pipette, 2 mL	Starlab	Cat# E4861-002
Stopper conical, 2 holes, upper diameter 45 mm, lower diameter 34 mm, height 45 mm	VWR	KART3848
Stopper conical, 2 holes, upper diameter 45 mm, lower diameter 34 mm, height 45 mm	VWR	KART3832
Stainless steel 19G 40 mm needle	BD Microlance	301500

## Materials and equipment

### Tumor slice *ex vivo* culture media recipe

**Table:** This medium can be stored up to at least one month at 4°C

Reagent	Final concentration	Amount
Human serum	5%	25 mL
RPMI 1640 with phenol red	NA	442 mL
Anti-anti 100X	1X	5 mL
Pen/Strep 100X	1X	5 mL
ITS-A 100X	2X	10 mL
Hydrocortisone 50 μg/mL	0.5 μg/mL	5 mL
MEM vitamins 100X	1X	5 mL
Fetuin 1 mg/mL	12.5 μg/mL	3.125 mL
Human recombinant EGF 0.5 mg/mL	10 ng/mL	10 μL
BSA for cell culture	2 mg/mL	0.5 g
3,3′,5-Triiodo-L-thyronine 20 μg/mL	100 pM	Dilute stock 1:10, then add 8.41 μL
O-Phosphorylethanolamine 0.5M	10 μM	5 μL
Ethanolamine 98%	10 μM	Dilute stock 1:100 then add 15 μL
**Total**		500.163 mL

**CRITICAL:** Sodium hydroxide (1 M) used to prepare the 3,3′,5-Triiodo-L-thyronine stock solution is highly corrosive and should be handled with appropriate Personal Protective Equipment (PPE), including safety glasses. Ethanolamine 98% is also corrosive and should be handled with similar care.

**Table:** 

Imaris analysis workstation specificationsSupplier	DELL
Processor	Intel Xeon CPU E5-1650 v3 3.5 GHz
Operating system	Windows 10 Enterprise, 64-bit
RAM	128 GB
Graphic card	Nvidia Quadro P4000

## Step-by-step method details

### Preparing and slicing the tumor sample

**Timing: 3 h**

This major step consists of producing tumor slices from the collected fresh tumor sample. This involves cutting the tumor sample into “sticks”, embed those in agarose, before slicing them with a Vibratome VT1200S. While we only use the Vibratome for this protocol, we believe it is possible to use other precision cut machines.

1. Cut the tumor into sticks.
  ***Note:*** This step should be performed in cold PBS. However, we have found it more practical to work with the PBS containing dish directly on bench, under a cell culture hood rather than on ice. If required, replace the PBS with fresh and colder PBS during the procedure.
  a. After collection and transport to the laboratory, place the tumor in a 150 mm petri dish, filled with cold sterile PBS, under a tissue culture hood.
  b. Keeping the tumor in cold PBS, assess the stiffness of the tissue using forceps. If needed, use the scalpel to cut some of the tissue and inspect inside the specimen. Stiff tissue will slice easier than soft tissue.
    i. If possible, remove adipose tissue as this is impossible to slice with the Vibratome.
    ii. Fibrotic tissue (if with low cellularity) can be elastic and this makes slicing difficult. If possible, avoid those elastic areas.
    iii. Remove any cauterized areas.
    iv. Remove areas of obvious necrosis unless these are of particular interest for the experiment.
  c. Still keeping the tumor in cold PBS, using the scalpel, cut the tumor specimen into sticks. Sticks should not be longer than 10 mm and not larger than 4 × 4 mm. Ideally, aim for 8–10 mm in length and 3 × 3 mm in width (Figure 2A).

**Figure 2:**
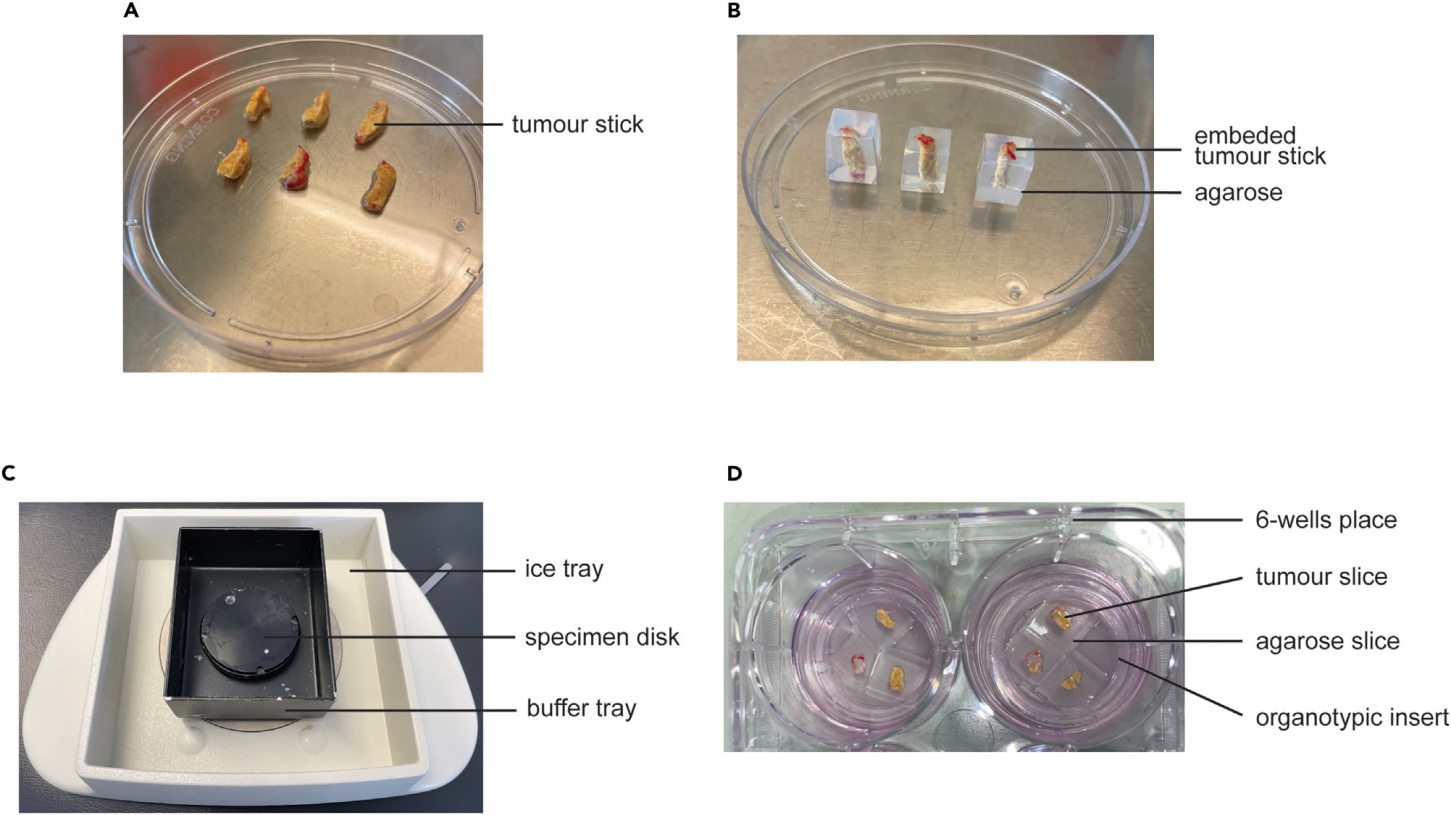
Example of tumor sticks, before and after agarose embedding and post slicing, related to steps 1 to 5 (A), high grade serous ovarian omental metastasis tumor cut into sticks 8–10 mm in length and 3 × 3 mm in width. (B), tumor sticks embedded in 5% low melting point agarose-PBS and cut into cubes leaving 2–3 mm on each side of the embedded tumor stick. (C), Vibratome tray containing the buffer tray and the specimen disk. D, Agarose slices containing tumor slices immediately transferred to a 6-wells plate containing organotypic inserts after slicing.
  d. Transfer the cut sticks to another petri dish containing fresh cold sterile PBS to rinse them and keep them on ice until ready to embed.
    **CRITICAL:** The wider the stick, the larger the slice. Large tissue slices are more likely to flap out of the agarose slice. In addition, a too long stick might bend when sliced at the top.
    While sticks are the easiest shape to quickly produce slices of regular surface area, it is not always possible to cut such a shape or such dimensions. Therefore, the shape and size of the pieces of tumor that are embedded in agarose must be adapted to the specimen. In addition, very small samples can also be sliced. For example, we have embedded and successfully sliced very small tumors resected from mouse models that measured 2 mm in length and 1 × 1 mm in width and depth. These should be embedded without pre-cutting.
    Methods video S1. Embed tumor sticks, related to step 3
    Methods video S2. Trim the agarose cube, related to step 3
    Methods video S3. Mount the agarose cube on specimen disk, related to step 4
    Methods video S4. Mount blade on the Vibratome, related to step 5
    Methods video S5. Set Vibratome’s slicing parameters, related to step 5
    Methods video S6. Slice tumor sticks, related to step 5
2. Prepare the 5% agarose gel in PBS, using low gelling point agarose.
  a. Prepare 10 mL per stick.
  b. For 10 mL: weigh 0.5 *g* of low gelling point agarose and add 10 mL sterile PBS.
  c. Microwave until bubbles appear and stir thoroughly. Repeat until agarose is dissolved.
  d. Set the agarose aside in an incubator at 37°C until use.
3. Embed tumor sticks.
  a. Prepare disposable embedding molds, one per stick. Label them if necessary.
  b. Quickly dab the tumor sticks on a paper tissue to remove the excess PBS. Avoid prolonged contact with the tissue to not dry the stick.
  c. Place the sticks on a dry petri dish lid, on ice.
  d. Retrieve the agarose from the incubator and check the temperature. It should not be warmer than 37°C. If it is, let it cool down further at room temperature.
  e. Embed one stick at a time (Methods video S1).
    i. Fill one embedding mold with agarose.
    ii. Using the forceps, place one stick in the gel, vertically, at the center of the mold.
    iii. Depending on tissue content, the tissue might sink or float. Correct the position of the stick in the mold, making sure it does not touch the edges.
    iv. Place on ice for 15 min.
      **CRITICAL:** We advise to start preparing the Vibratome (step 4) during this waiting time to minimize the time between when sticks have been embedded and trimmed and when they are mounted on the Vibratome’s specimen disk.
      **CRITICAL:** If the tissue touches the edge of the mold, the tumor slice will not hold in the agarose and will easily escape when sliced.
  f. Once the agarose is fully set, carefully break open the embedding mold, without damaging the agarose.
  g. Using a scalpel, trim the agarose around the tumor stick so that there is at least 2–3 mm of agarose around the stick (Figure 2B and Methods video S2).
    **CRITICAL:** Aim for the shape of the agarose block to be close to cubic post trimming. If the shape of the block is too long and too narrow, there is a risk of the gel bending upon contact with the advancing Vibratome blade. This will compromise the slicing and can result in the block ripping from the specimen disk. If the tumor stick is long, it is therefore advised to make the block’s shape as wide as it is long (close to cubic) as this provides the best stability.
  h. Leave the blocks in a dry petri dish, on ice, until ready to mount on the Vibratome’s specimen disk.
    **CRITICAL:** Ensure this step does not last more than 2 min to ensure the tumor sticks do not dry out.
4. Mount the blocks (Methods video S3).
  a. Remove the ice tray from the Vibratome. Leave the buffer tray in the ice tray (Figure 2C).
  b. Fill the ice tray with ice.
  c. Remove the specimen disk from the buffer tray.
  d. Place the ice tray (with the buffer tray in) at 4°C until the blocks are mounted.
  e. Tilt the Vibratome’s blade holder upwards using the size 3 Allen key (provided with the Vibratome).
  f. Carefully place a disposable razor blade in the blade holder. Tighten the holder to secure the blade.
  g. Using the small nozzle of the cyanoacrylate glue tube, spread glue in the shape of a small square the size of a block on the specimen disk.
  h. Using the forceps, place the block on the glue, gently moving it so that the glue spreads to the whole surface of the block’s base.
  i. If necessary, add a small drop of glue on each side of the block (where the block meets the specimen disk) to secure it further.
    ***Note:*** It is possible to place several blocks on the specimen disk. If the blocks are placed side by side, it allows slicing of multiple blocks simultaneously (provided they are from the same specimen). We have put up to three blocks side by side. This may sometimes require trimming the block into smaller cubes.
    **CRITICAL:** Do not place the block on the edge of the specimen disk or on top of any of the two specimen disk’s holes, as the Vibratome blade doesn’t reach all the way to the edges.
  j. Place the specimen disk back in the buffer tray.
  k. Fill the buffer tray with ice cold PBS. The agarose blocks should be completely submerged.
5. Slice the specimen using the Vibratome.
  a. Retrieve the 6-well plates containing the *ex vivo* tumor slice media and organotypic inserts.
  b. In addition, save some tissue sections for histology analysis or immunohistochemistry by placing them in 24-well plates containing formalin.
  c. Place the ice tray back on the Vibratome, ensure it is secure.
  d. Lower the blade so that only the left white line is visible (Methods video S4). This puts the blade at the proper angle with the top of the agarose block.
    **CRITICAL:** Dot not set the blade parallel to the agarose block as this will cause damage to the tissue
  e. Set the Vibratome on blade speed 0.3 mm/s, amplitude 1.5 mm and feed 350 m. Using automatic – continuous mode is recommended.
  f. Raise the stage so that the agarose block is just under the blade.
  g. Set start and finish positions.
  h. Moving the blade manually, set the start and finish blade position so that slicing starts approximately 1 mm before and finishes 1 mm after the agarose block (Methods video S5).
    ***Note:*** This ensures better quality slicing and accounts for potential bending of the block during slicing.
  i. Pull the blade back so that it is not above the block anymore.
  j. Raise the stage so that the edge of the blade is now lower than the top of the block. Ideally, the edge of the blade should be levelled with the top of the tissue.
  k. Start slicing.
    i. Discard the first slice.
    ii. Carefully pick up the following slices with a pair of forceps (Methods video S6).
    iii. Place them on the organotypic inserts, in the prepared 6-wells plate with *ex vivo* slice media. Place up to three slices per well (Figure 2D).
  l. Directly fix some slices in the 24-wells plate prepared with formalin for analysis by histology methods.
    i. With forceps, gently grab the tumor slice.
    ii. Shake the tumor slice off the agarose slice.
    iii. Place the tumor slice in formalin.
    iv. Rinse and wipe the forceps before returning it to the buffer tray to avoid contamination with formalin.
  m. Once slicing is done, return the 6-well plate to a tissue culture hood and add a stainless-steel ring washer on top of each slice (Methods video S6). Ensure the ring surrounds the tissue but does not cover it.
  n. Add 100 μL of media inside the steel ring (pipette directly from the well the slice is in).
  o. Place the 6-wells plate containing the tumor slices back in the tissue culture incubator at 37°C for at least 10 min before staining.
    **Pause point:** At this stage it is possible to leave the tumor slice *ex vivo* for up to 24 h at 37°C without loss of viability.^1^ This provides an opportunity to incubate the slices with different treatments. If slices are analyzed at different time points, we recommend fixing slices from each time point and condition in 10% formalin to use as control with histology.

### *Ex vivo* tumor slice immunofluorescence staining and mounting

**Timing: 60 min**

This major step consists of staining and mounting the *ex vivo* tumor slices for fluorescent live imaging. This includes blocking the Fc receptor, staining malignant cells, the extracellular matrix (fibronectin), CD8^+^ T cells and myeloid cells with antibodies directly conjugated to fluorochromes; and mounting the stained slices in an imaging dish containing unsupplemented phenol red-free RPMI 1640 media.

6. Block Fc Receptors.
  a. After the minimum 10 min incubation time and a maximum of 24 h at 37°C, transfer the slice to a 30 mm petri dish using forceps and place the stainless-steel washer back on top of the tissue.
  b. Add 20 μL undiluted human TruStain inside the ring, on top of the tissue.
  c. Incubate 15 min at 37°C.
  d. Remove the blocking solution.
7. Immunofluorescence staining.
  a. Prepare the antibody mix.
    i. Each antibody dilution must be empirically optimized. We recommend 50 μg/mL as a starting concentration. Table 1 shows the antibody mix we have used:

**Table 1:** Antibody panel for real-time imaging of myeloid and CD8^+^ T cells in human ovarian tumor slices

Target	Marker	Dye	Dilution	Manufacturer	Catalogue number
Cytotoxic T cells	CD8	Alex Fluor 647	1:10	BioLegend	344725
Myeloid cells	CD11b	Alex Fluor 594	1:10	BioLegend	301340
Malignant cells	EpCAM	Alex Fluor 488	1:10	Thermo Fisher Scientific	53-8326-42
Extracellular matrix	Fibronectin	Alex Fluor 405	1:10	Novus biologicals	NBP2-34633AF405
    ii. Antibodies are diluted in unsupplemented phenol red-free RPMI 1640.
    iii. When optimizing, include a single staining with each antibody.
    iv. Also include a slice that will not be stained to assess tissue autofluorescence.
  b. Add 20 μL of antibody mix (or phenol red-free RPMI 1640 if assessing autofluorescence) inside the stainless-steel washer, on top of the tissue.
  c. Incubate slices with antibody mix for 15 min at 37°C.
  d. Remove antibody mix.
  e. Wash slices with phenol red-free RPMI 1640.
    i. Add 40 μL phenol red-free RPMI 1640 on top of the tissue, pipette up and down and discard the media.
    ii. Add another 40 μL of fresh phenol red-free RPMI 1640.
    iii. Incubate 2 min at 37°C.
    iv. Discard washing media and replace with 40 μL of fresh phenol red-free RPMI 1640.
    v. Incubate 2 min at 37°C.
    vi. Discard washing media.
8. Mount the slices (Methods video S7).
  a. Using forceps, remove the stainless-steel washer, carefully flip and transfer the slice into a 30 mm glass bottom dish.
    **CRITICAL:** It is important to flip the slice, as the stained side must face down for imaging with an inverted microscope. If using an upright microscope, do not flip the slice.
    ***Optional:*** If necessary, before transferring the slice, add 100 μL of phenol red-free RPMI 1640 on top of the slice and another 100 μL around it to make it easier to pick up.
    ***Optional:*** It is possible to mount up to four slices together in the same dish (recommended if imaging multiple slices). For this, the agarose around the tissue must be gently trimmed using a scalpel, in order for all slices to fit on the glass bottom of the imaging dish. In addition, we recommend trimming the side of the agarose slice that faces other slices to ensure that all slices fit inside the tissue slice anchor. As the mounting of multiple slices on the same dish can take time, ensure that all slices stay hydrated by adding 20 μL phenol red-free RPMI 1640 to them after transferring them to the imaging dish.
  b. Add the tissue slice anchor on top of the tissue. Ensure it is positioned in such a way that it surrounds all tissue slices and does not lie on top.
    **CRITICAL:** First dip the anchor in phenol red-free RPMI 1640. When the anchor is dry, it is hydrophobic and is likely to float when filling the imaging dish with media, which in turn can move the slices.
  c. Slowly add 1 mL phenol red-free RPMI 1640 (imaging media) on top of the slice(s) with a P1000. Ensure neither the tissue slice(s) nor the anchor move in the process. Place the lid back on top of the imaging dish before transporting to the microscope.

Methods video S7. Transfer slice to imaging dish, related to step 8

### *Ex vivo* tumor slice real-time imaging

**Timing: 2 h**

9. Set up the medium perfusion.
  a. Set the chamber to 37°C and 5% CO_2_.
  b. Warm up 250 mL phenol red-free RPMI 1640 (imaging media).
  c. Enrich imaging media with oxygen.
    i. Transfer warmed up media to a conical flask with an air outlet and add a stopper with two holes (Figure 1).
    ii. Place the flask with imaging medium in the pre-heated imaging chamber.
    iii. Clean the 2 mL pipette (tubing end 2) connected to the carbogen source with 70% ethanol and air dry thoroughly.
    iv. Through one hole, introduce the pipette connected to the source of carbogen (tube end 2). Plunge the tubing in the imaging media.
    v. Open the carbogen regulator (0.1 bar) to bubble the media.
  d. Clean peristaltic pump tubing (Figure 1).
    i. Attach two bent 19G 40 mm needles to the in flow bioprene tubing end 4 and outflow end 5 of the peristaltic pump tubing.
    ii. Plunge the 2 mL pipette connected to tube end 3 along with both needles (connected to their respective ends) in 70% ethanol.
    iii. Circulate 70% ethanol through both channels of the pump for 3 min at full speed (100 rpm).
    iv. Take tube end 3 and the needle attached to tube end 5 out of the ethanol and run the pump at full speed to empty all tubes of ethanol.
    v. Plunge the 2 mL pipette connected to tube end 3 along with both needles (connected to their respective ends) in sterile double distilled water.
    vi. Circulate water through both channels of the peristaltic pump for 5 min at full speed.
    vii. Empty the tubing of all remaining water.
  e. Set the oxygenated media perfusion in the imaging dish.
    i. Introduce the pipette connected to tubing end 3 of the in flow channel in the second hole of the conical flask containing the imaging media.
    ii. With both needle ends over an empty beaker, run the pump on maximum speed to fill the tubing with oxygenated imaging media (until the medium comes out of the in flow needle).
    iii. Stop the pump and place needles inside the temperature-controlled chamber, tips in the imaging dish, but not touching the slice nor the anchor.
    iv. Secure needles in place. We use a magnetic petri dish hold down (provided with the 35 mm holder stage insert), which in absence of the petri dish lid secures the needles to the dish (Figure 3).

**Figure 3:**
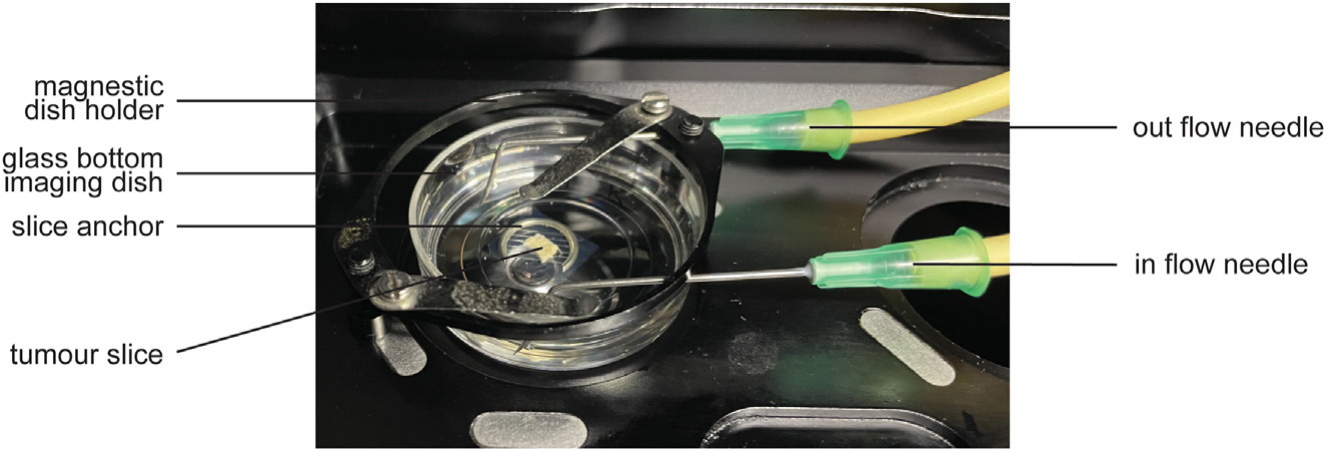
Tissue slice imaging setup, related to step 9 In a 30 mm glass bottom Petri dish, a tumor slice, still encased in agarose gel is held down by a slice anchor and covered in 3 mL imaging media. Two bent needles, respectively connected to the in flow and out flow sections of bioprene tubing, are secured in the dish using a magnetic dish holder, with the dish’s lid removed.
    v. Run the pump on maximum speed for 20 s to replace the imaging medium with oxygenated imaging medium.
    vi. Set the pump on 6 rpm (flow approximately 1 mL/min).
    vii. Let the perfusion run for at least 10 min before recording time-lapse.
10. Image the tumor slice.
  a. Record images of multiple fields. We recommend recording 10 fields, taking care of choosing areas representative of the tissue.
    i. Visualize each channel in the following order: blue (DAPI), green (GFP), red (RFP) and far red (Cy5).
    ii. Adjust gain or exposure for each channel. If comparing multiple conditions use the same setting for all.
    iii. Acquire within 100 μm depth, z step = 5 μm.
  b. Choose at least two areas per slice to record the time-lapse and record their positions. The time-lapse for both positions will be acquired simultaneously. If possible, select an area containing CD8^+^ T cells, myeloid cells, malignant cells and fibronectin.
    i. Restrict depth of z stack to 40–70 μm and set a z step of 5–7 μm.
    ii. Set the microscope to acquire one image every 30 s for 30 min (a total of 61 time frames).
      **CRITICAL:** For accurate cell tracking, the timeframe should not exceed 30 s. This requires optimizing the exposure for each channel, the number of fields acquired in the same timelapse, and the number of z steps. Decreasing exposure, reducing z stack size and increasing z step size helps shortening the acquisition time for each timeframe. The signal intensity lost by decreasing exposure can be compensated by increasing the laser power. We routinely analyze four fields in one single time lapse, with four channels, z step 7 μm, z-stack size 40 μm.
  c. During the first 3 to 4 time frames, visualize the beginning of the time-lapse and ensure there is no drifting. If needed, abort time-lapse and secure the slice further by moving the anchor.
11. Saving the time-lapse recording.
  a. Once time-lapse is completed, visualize each z slice.
    ***Note:*** It may be required to crop the top or bottom slice if the tissue displays signs of cells death (when all cells are completely static on the top or bottom z slice but there is movement in other z slices), which could be caused by the slicing. The rest of the tissue should not be affected.
  b. Create a maximum intensity projection for the analysis.
  c. Add a scale bar and save the projection as a new file. Troubleshooting 1.
    To reduce drifting, avoid damaging the agarose slice around the tissue. The agarose helps secure the slice in place. In addition, make sure the anchor’s threads are above the tissue itself and in contact with it to hold it down. Finally, avoid having the needle tips too close to the slice so that the medium flow does not dislodge it. Troubleshooting 2.
    Photobleaching is a rare occurrence in a 30 min time-lapse. However, should it occur, limit as much as possible exposure to light by working with minimal lighting during the staining procedure and when mounting and setting up the slices in the microscope. Finally, reducing the laser power will decrease photobleaching too. This might in turn decrease the signal’s intensity. If increasing the exposure is not possible to compensate this, it is possible to use binning 2 × 2 to increases the sensitivity and therefore the intensity. Note however that this reduces the resolution four times.

### Tracking the CD8^+^ T and myeloid cell movements

**Timing: 45 min per film**

12. Correcting the drift.
  a. Open the maximum intensity projection file with Bitplane Imaris.
  b. Visualize the recording and assess if the tissue is drifting. If not, proceed to step 13. If it is, use the Fiji correct 3D drift plugin (steps 12.c to 12.g).
  c. Determine which channel should be used as a reference point.
    **CRITICAL:** The reference channel must not display any movement; this includes moving cells or particles. If none of the channels are fully static, continue with step 12.d, otherwise go to step 12.e.
  d. If no channels are fully static, use an object (cell or particle) that does not display any movement.
    i. Using the Spot function in Imaris, create a spot for this object and track over time.
      ***Optional:*** Creating a surface instead of a spot also works and the user should try both to decide what works best for their analysis
    ii. Create a very small region of interest around the chosen cell or particle.
    iii. Set the diameter to the size of the chosen cell or particle and use background subtraction.
    iv. Ensure the quality threshold selected only detects the chosen cell or particle.
    v. Set the tracking algorithm to a low maximum speed to ensure the detected trajectory is accurate.
    vi. Mask the resulting Spot to a new channel. This creates a new channel containing only the Spot.
    vii. Select the same channel used to detect the Spot.
    viii. Ensure the option to duplicate channel before applying mask is selected.
    ix. Select Constant inside/outside and set voxel outside spot to 0.
    x. Apply to all time points.
    xi. Click OK.
    xii. Select this new channel when choosing the reference channel in the Fiji drift correction.
  e. If drift is > 3 μm, leave all options unticked.
  f. If drift is < 3 μm, use the multi time scale option.
    ***Optional:*** For very short drifts, drift correction could benefit from using Sub pixel drift correction and Edge enhanced options.
  g. In Imaris, visualize the recording again. If there is residual drift that needs to be corrected, perform steps 12.c to 12.g again.
13. Track the movement of CD8^+^ T cells.
  a. Create spots.
  b. If drift correction was needed, select analyze ROI option and crop the edges of the recording accordingly.
  c. Do not use the region growing option.
  d. Select the CD8 channel and set the diameter to 7 μm, do not select the estimated z diameter option.
  e. Use the background subtraction option and set a threshold that maximizes the detection of CD8^+^ T cells without detecting background.
  f. Set the tracking algorithm.
    i. Select the Brownian Motion algorithm.
    ii. Maximum distance must be optimized by the user, but for T cell we recommend starting between 10 and 15 μm.
    iii. Set Max Gap Size to 3 and disable the Fill gap with detected object option.
      **CRITICAL:** Due to the relatively high speed of T cells, Imaris sometimes makes mistakes when tracking cells. Therefore, once the tracking is finished, visualize the recording and manually delete tracks that are incorrect.
  g. In the statistics tab, select Detailed export all statistics to file. This creates a folder containing all the statistics calculated by Imaris, in .csv format.
    ***Note:*** When naming the folder, we recommend using “_” for easier automation of the analysis in R (see quantification and statistical analysis). All files in the folder will therefore be named “__[statistic].csv”. We recommend subsequently renaming the folder with a name of the analyzed recording file and the cell type analyzed (for example: “CD8_slice1_a”).
14. Track the movements of myeloid cell.
  a. Follow step 13.a to 13.g again, but this time select the CD11b channel and set diameter to 10 μm.
  b. Rename the exported folder including the name of cells analyzed (for example: “CD11b_slice1_a”).

***Note:*** we have successfully analyzed recordings that contained hundreds of cells without being limited by the computer’s capacity. The computer’s specifications are noted in the materials and equipment section.

## Expected outcomes

This protocol builds on techniques developed to study the migration of cytotoxic T cells in real-time in human lymph nodes and tumors *ex vivo*.^2^^,^^3^ We have further developed this so that it now allows the study of myeloid cells.^1^

In addition, prior to imaging, tumor slices can be maintained viable in culture with the tumor slice media for 24 h *ex vivo*, providing the ability to investigate the impact of treatments on CD8^+^ T and myeloid cell movements.^1^

**Figure 4:**
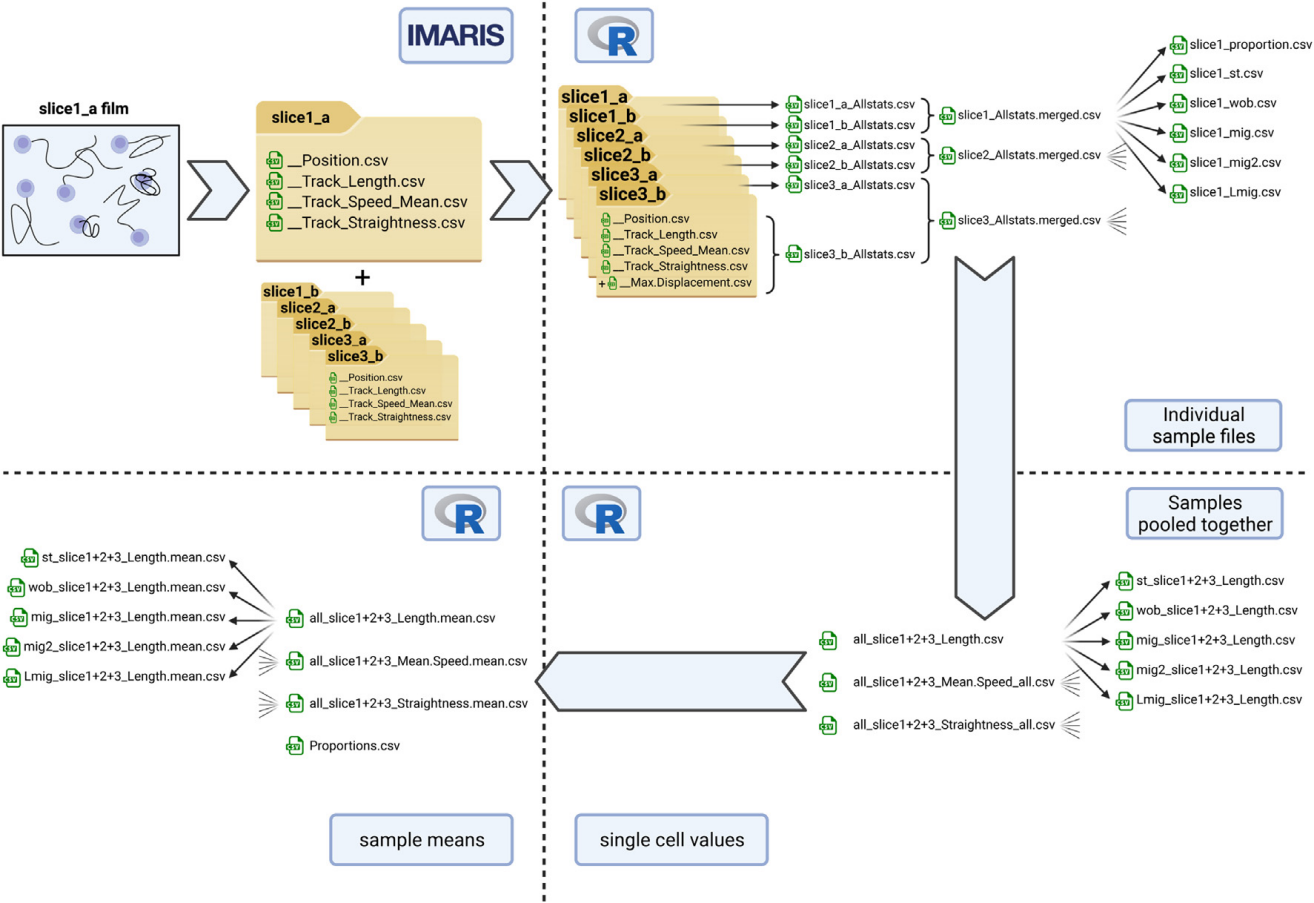
Cell behavior data aggregation and analysis, related to quantification and statistical analysis For each slice there should be two films recorded and analyzed by Imaris. Each film analyzed produces an output folder containing all the movement metrics (“statistics”), including the “__Position.csv”, “__Track_Length.csv”, “__Track_Speed_Mean.csv”, “__Track_Straightness.csv” files, used to split the different behavior categories^1^ and quantify cell movements. Once all the Imaris output folders put together in one folder (working directory), the R script computes the maximum displacement (“TDisMax.csv”), and merges it with the track length, mean speed and straightness data on a cell-by-cell basis. The output is the “Allstats.csv” file. The script then aggregates the films of the same slice together into one “Allstats.merged.csv” file, splits the different populations (static, st; wobbling, wob; migrating, mig; long migrating, Lmig and migrating + long migrating, mig2), producing a file with single cell value for each population and each slice. It then computes the proportions of cells belonging to each categories producing a corresponding file for each sample. The script subsequently aggregates samples together and produces one file per movement parameter of interest (Track Length, Mean Speed and Straightness) containing single cells values for all samples together. Subsequently, the script also splits the different behavior populations producing one file per movement parameter and population. Finally, the scrip computes the sample (slice) mean for each parameter, produces a file containing a single value for this parameter for each sample and splits the behavior populations, producing a file containing the sample mean values for each parameter and population. The script also computes the proportion of cells belonging of each cell behavior population.

Finally, this protocol facilitates cell behavior analysis. We previously identified four distinct cell behavior categories: static, wobbling, migrating and long migrating. This protocol facilitates the aggregation of movement data, splitting the four distinct behavior categories^1^ and producing multiple convenient outputs (Figure 4).

This protocol provides movement data for two immune cell types, at multiple time points and the possibility to compare multiple conditions. Tumor slices can therefore be stained and imaged shortly after slicing or after up to 24 h of *ex vivo* culture. For this, users maintain the slices in the tumor slice media *ex vivo* until they need to image it. They then proceed to stain and image the slice as per protocol steps 6–11 . The data generated can be processed in a semi-automated manner to study, together or separately, multiple behavioral cell populations.

## Quantification and statistical analysis

The R script we developed provides a simple way to aggregate cell behavior data and analyze the different behavior populations (as described in Laforets et al.^1^), together or separately. It produces multiple outputs, for individual samples or for all samples together and with either single cell values or sample means (Figure 4). We have the Imaris outputs of 3 slices (6 films) for the user to test the R script. These are located in the “test” folder. This publication also includes the “Aggregate_stats.R” R script file. We recommend the user directly opens this file in R studio rather than copying and pasting lines of code from this article.

1. Prepare the working directory (folder). Figure 4.
  a. In a single folder, place all the output folders from the Imaris analysis of CD8^+^ T cell movements. There should be one folder for each film analyzed. Ideally there should be two films (therefore two folders) per slice imaged. However, it is possible to run the script even if some slices have only one film (one folder).
  b. Ensure that all folders contain the movements statistics .csv files named “_[statistic].csv”.
  c. Ideally, there should be two films per slice, therefore two folders per slice. If so, ensure that the two films of the same slice have the same name with different suffixes (for example: slice1_a and slice1_b).
2. Run the script.
  a. Open the R script in R Studio.
  b. Run lines 12 and 13 to install and load the required packages.
    >install.packages("tidyverse")
    >library(tidyverse)
  c. Modify the file path for the working directory on lines 16 and 17 so that it refers to location the folder containing the data you wish to analyze.
    >setwd("C:/ ")
    >wdir <- "C:/"
  d. Run lines 21 to 23 and check that the output contains all the film folders of interest.
    >folders <- list.dirs(path = wdir)
    >folders <- folders[-1]
    >folders
  e. Run line 27 to 89. This generates an “Allstats” .csv file for each film folder.
    >for (folder in folders) { # iterates the script in all input folders
    > ## Load .csv files containing the statistics of interest for one film, with only the track number and stat columns
    > dir.name <- basename(folder) # Gets the input folder name
    > filepath <- paste(wdir, dir.name, sep = "/") # Creates a generic file path for input files
    >
    >
    > ## Calculate the maximum displacement and create a .csv file for it
    > filepath.Pos <- paste(filepath, "__Position.csv", sep = "/") # Create a file path for the Position.csv file
    > Positions <- read.csv(filepath.Pos, skip = 3, fill = TRUE, header = TRUE)[,c(1,2,7,8)] # Read Position.csv including only the necessary columns
    >
    > IDs <- unique(Positions["TrackID"]) # Retrieves a list of all Track IDs
    >
    > Positions.arranged <- arrange(Positions, TrackID)
    >
    > # Subset the data for each track
    > listx <- list()
    > for (i in seq(1, nrow(IDs))) {
    >  listx[[i]] <- subset(Positions.arranged, TrackID == 1000000000 + i -1)
    > }
    >
    > # Create a list identical to listx but with only t0. This is used as the initial position for calculating the displacement at each time point
    > list.t0 <- list()
    > for (i in seq(1, nrow(IDs))) {
    >  list.t0[[i]] <- subset(Positions.arranged, TrackID == 1000000000 + i -1)
    >  list.t0[[i]] <- list.t0[[i]][1,] # Retains only the time point 1 row
    >  list.t0[[i]] <- list.t0[[i]][rep(seq_len(nrow(list.t0[[i]])), nrow(listx[[i]])), ] # Fills the data with repetition of time point 1
    > }
    >
    ># Calculate the displacement of each cell at each time point
    > for (i in seq(1, nrow(IDs))) {
    >  listx[[i]]$Dis <- sqrt((listx[[i]][1]-list.t0[[i]][1])ˆ2 + (listx[[i]][2]-list.t0[[i]][2])ˆ2)
    > }
    >
    > # Creates a list with only the maximum displacement observed for each track
    > list.DisMax <- list()
    > for (i in seq(1, nrow(IDs))) {
    >  list.DisMax[[i]] <- data.frame(max(listx[[i]][4]), max(listx[[i]][5]))
    > colnames(list.DisMax[[i]]) <- c("ID", "MaxDis")
    > }
    >
    > TMaxDis <- do.call("rbind", list.DisMax) # Binds all the maximum displacement data frame in the list into one.
    >
    > write.csv(TMaxDis, paste(filepath, "__Max_Displacement.csv", sep = "/"), row.names = FALSE) # write the file in the corresponding input folder
    >
    > ## Create filepaths for each required statistic file
    > filepath.Length <- paste(filepath, "__Track_Length.csv", sep = "/")
    > filepath.Speed <- paste(filepath, "__Track_Speed_Mean.csv", sep = "/")
    > filepath.Straightness <- paste(filepath, "__Track_Straightness.csv", sep = "/")
    >
    > ## Read all required statistic files
    > TLength <- read.csv(filepath.Length, skip = 3, fill = TRUE, header = TRUE)[,c(1,4)]
    > TSpeed <- read.csv(filepath.Speed, skip = 3, fill = TRUE, header = TRUE)[,c(1,4)]
    > TStraightness <- read.csv(filepath.Straightness, skip = 3, fill = TRUE, header = TRUE)[,c(1,4)]
    >
    > ## Merge the required statistic files according to the ID columns
    > Allstats <- Reduce(function(x, y) merge(x, y, by = "ID", all=TRUE), list(TLength, TSpeed, TStraightness, TMaxDis)) # The name "Allstats" refers all to the statistics of interest used to split the different behavioral categories, but it does not actually contain all the statistics produced by Imaris
    > head(Allstats) # Check the Allstats output
    > dim(Allstats)
    >
    >## Write a .csv file containing all statistics of interest for the corresponding film
    > filename1 <- paste(dir.name, "Allstats.csv", sep = "_")
    > write.csv(Allstats, paste(wdir, filename1, sep = "/"), row.names = FALSE)
    >}
    >
  f. If you have only one film for some of the slices (non-paired):
    i. Create a folder (at any location).
    ii. Move the “All stat.csv” files corresponding to the non-paired film to the newly created folder.
    iii. Rename the non-paired “All stat.csv” files as “All stat.merged.csv”
  g. Run line 94 to 107.
    >files <- list.files(wdir, pattern = "∗.csv", full.names = FALSE) # Get a list of all Allstats files
    >files
    >ldf <- sapply(files, read.csv, simplify = FALSE, USE.NAMES = TRUE) # Read all Allstats files and store in a list
    >ldf # Check list output
    >
    ># Merge Allstats files from two movies of the sample: iterate every two files
    >for (i in seq(1,length(ldf)-1, 2)){ # Iterate every two file, stop at the next before last file
    > merged <- rbind(ldf[[i]], ldf[[i+1]]) # Bind the two Allstats dataframe one on top the other
    > write.csv(merged, gsub("a_Allstats.csv", "Allstats.merged.csv", names(ldf[i])), row.names = FALSE) # Write the merged output
    > name <- paste(gsub(".csv", "", names(ldf[i])), "merged", sep = ".") # Name the output data frame for easier handling in R
    > assign(name, merged)
    >}
    >
  h. Place the renamed non-paired files back in the working directory.
  i. Run the rest of the script (lines 117 to 752, supplemental document).
3. Check the output.

In the working directory, ensure that all outputs are present.

***Note:*** There are two types of outputs: movement statistic for each individual cell, and the mean per slice (sample mean). The working directory should now contain these two types of .csv files for the trajectory length, mean speed and straightness; for the overall population (all), static cells (st), wobbling cells (wob), migrating cell (mig), long migrating cells (Lmig) and migrating + long migrating cells pooled together (mig2). The working directory should also contain the proportion file.

4. Repeat step 1–3 for CD11b cell movement data.
5. Plotting the movement statistics.
  How to plot the data depends on the user’s hypothesis. We would however highlight two different ways of doing so.
  a. One is to plot as histograms (with individual data points) a movement statistic (i.e., trajectory length), with data points being the mean of all cells for each slice.
  b. Another way is to plot the statistic value for each individual cell.
    **CRITICAL:** Method a. ignores differences in cell numbers between conditions or how heterogeneous a cell population is. It gives more weight to individual samples. Method b. instead gives more weight to individual cells. It also drastically increases the number of data points. As a result, when comparing conditions, statistical tests are more likely to show significant differences, despite sometimes limited difference. To compensate this, we strongly advise taking into account either the absolute difference between conditions or express this difference in percentage of the control mean. This will add depth to the results and help the user determine the biological significance of their results.
6. Statistical tests.
  a. We encourage the users to assess the normality of the distribution of their data before deciding which test to choose to compare conditions.
  b. So far, we have chosen the parametric t-test both for comparing two conditions using method a. and method b. If comparing together three on more conditions, we have used the parametric one-way ANOVA.

## Limitations

This protocol may not be suitable for all tumor specimens. Particularly, tumors with high fibrosis and low cellularity that are elastic make slicing imprecise, sometimes impossible. In addition, the low cellularity renders imaging of immune and malignant cells impossible. Tumors with very high fat content are also difficult to slice. Finally, some tissues (e.g., lung) also display autofluorescence, particularly in the green channel, which makes imaging in this channel difficult.

## Troubleshooting

### Problem 1

The Vibratome’s blade does not cut through the tumor stick smoothly and pushes it out of the agarose. Related to step 5.

### Potential solution

• Re-embed the tumor stick in fresh agarose.
• Ensure the agarose has had enough time to solidify (at least 15 min on ice).
• Reduce the blade speed to 0.2 mm/s, even 0.12 mm/s if needed.
• If this isn’t enough, reduce blade amplitude to 1 mm.

### Problem 2

The slice is moving during imaging. Related to steps 8 and 10.

### Potential solution

• Ensure the slice anchor is not upside down: the nylon threads should be in contact with the surface of the slice.
• Ensure the in flow needle isn’t too close to the slice, as it could cause it to move.
• Ensure there is enough medium in the dish to cover the slice and the anchor.
• If needed, before adding the anchor, add a drop of 2% low-melting point agarose on each side on the agarose slice to seal it to the glass. Take care that the agarose does not penetrate under the slice, however, as this would compromise imaging.

### Problem 3

Drifting observed in the time-lapse recording is not fully corrected. Related to step 12.

### Potential solution

• Repeat step 12 multiple times.
• For large drifts, it is often required to perform first a simple drift correction and then another with the multi-time scale option. Repeating the latter can help further reduce the drift.

### Problem 4

Difficulty choosing the (background subtraction) quality threshold for the detection of cells in Imaris’ tracking module, when different slices display different fluorescent intensities. Related to step 13.

### Potential solution

• We recommend ensuring good cell detection, even if that requires adjusting different thresholds for different films. The purpose of this technique is to assess cell movement and not the cell marker’s intensity.

### Problem 5

Not all slices have two time-lapse recordings (films), therefore this causes problems when running the R script. Related to Quantification and analysis.

### Potential solution

• Before running lines 94 to 107, move the “Allstats.csv” files that aren’t paired to a different folder and rename them with the “.merged” suffix. Run lines 94 to 107 and then place the renamed files back in the working directory. You can resume running the rest of the script.

## Resource availability

### Lead contact

Further information and requests for resources and reagents should be directed to and will be fulfilled by the lead contact, Frances Balkwill (f.balkwill@qmul.ac.uk).

### Technical contact

Technical questions on executing this protocol should be directed to and will be answered by the technical contact, Florian Laforêts (f.laforets@qmul.ac.uk).

### Materials availability

This study did not generate new unique reagents.

### Data and code availability

The published article includes all code generated during this study. In addition, all original code has been deposited at Zenodo https://doi.org/10.5281/zenodo.11093977 and is publicly available as of the date of publication.

## Supplemental information

Data S1. Remaining R code to automate cell behavior data analysis, related to quantification and statistical analysis step 2

Data S2. Entire R script in .R format, related to quantification and statistical analysis step 2

Data S3. Files for testing the R script, quantification and statistical analysis step 2
