# Supplementary material for: Protocol for real-time monitoring of CD8+ T and myeloid cell behavior in human high-grade serous ovarian cancer slices
Source: STAR Protoc. 2024 Jun 2;5(2):103102. doi: 10.1016/j.xpro.2024.103102 (PMC11179100; doi:10.1016/j.xpro.2024.103102)
Supplement: Data S1. Remaining R code to automate cell behavior data analysis, related to quantification and statistical analysis step 2 [file mmc1.pdf]

```
>files_Allstats <- list.files(wdir, pattern = "Allstats.merged.csv",
full.names = FALSE) # Get all merged Allstats.merged files
>files_Allstats
>ldf_Allstats <- sapply(files_Allstats, read.csv, simplify = FALSE,
USE.NAMES = TRUE) # Read all merged Allstats files and keep their names
in the list
>ldf_Allstats # Check the output
>length(ldf_Allstats)
>
>## Subset static, wobbling, migrating and long migrating populations,
and iterate this for each sample (each merged Allstats.merged file)
>for (i in 1:length(ldf_Allstats)){
>  st <- ldf_Allstats[[i]][(ldf_Allstats[[i]]$MaxDis<7) &
(ldf_Allstats[[i]]$Track.Length<13), ] # subset static cells
>  colnames(st) <- c("ID", "Track.Length", "Track.Speed.Mean",
"Track.Straightness", "Max.Displacement.Length")
>  wob <- ldf_Allstats[[i]][(ldf_Allstats[[i]]$MaxDis<7) &
(ldf_Allstats[[i]]$Track.Length>=13),] # subset wobbling cells
>  colnames(wob) <- c("ID", "Track.Length", "Track.Speed.Mean",
"Track.Straightness", "Max.Displacement.Length")
>  mig <- ldf_Allstats[[i]][(ldf_Allstats[[i]]$MaxDis>=7) &
((ldf_Allstats[[i]]$MaxDis<25) | (ldf_Allstats[[i]]$Track.Length<40)),]
# Subset migrating cells
>  colnames(mig) <- c("ID", "Track.Length", "Track.Speed.Mean",
"Track.Straightness", "Max.Displacement.Length")
>  mig2 <- ldf_Allstats[[i]][(ldf_Allstats[[i]]$MaxDis>=7), ] # Subset
migrating + long migrating cells together
>  colnames(mig) <- c("ID", "Track.Length", "Track.Speed.Mean",
"Track.Straightness", "Max.Displacement.Length")
>  Lmig <- ldf_Allstats[[i]][(ldf_Allstats[[i]]$MaxDis>=25) &
(ldf_Allstats[[i]]$Track.Length>=40),] # Subset long migrating cells
>  colnames(mig) <- c("ID", "Track.Length", "Track.Speed.Mean",
"Track.Straightness", "Max.Displacement.Length")
>  Prop.types <- data.frame((nrow(st)*100/nrow(ldf_Allstats[[i]])),
(nrow(wob)*100/nrow(ldf_Allstats[[i]])),
(nrow(mig)*100/nrow(ldf_Allstats[[i]])),
(nrow(Lmig)*100/nrow(ldf_Allstats[[i]]))) # Calculate the porportion of
each behaviour category
>  colnames(Prop.types) <- c("St", "Wob", "Mig", "Lmig")
>
>  # Set proper file names
>  name.st <- gsub("_Allstats.merged.csv", "_st",
names(ldf_Allstats[i]))
>  name.wob <- gsub("_Allstats.merged.csv", "_wob",
names(ldf_Allstats[i]))
>  name.mig <- gsub("_Allstats.merged.csv", "_mig",
names(ldf_Allstats[i]))
>  name.mig2 <- gsub("_Allstats.merged.csv", "_mig2",
names(ldf_Allstats[i]))
>  name.Lmig <- gsub("_Allstats.merged.csv", "_Lmig",
names(ldf_Allstats[i]))
>  name.Prop.types <- gsub("_Allstats.merged.csv", "_Proportions",
names(ldf_Allstats[i]))
```

```
>  # Write .csv output for each behaviour category
>  write.csv(st, paste(name.st, ".csv", sep = ""), row.names = FALSE)
>  write.csv(wob, paste(name.wob, ".csv", sep = ""), row.names = FALSE)
>  write.csv(mig, paste(name.mig, ".csv", sep = ""), row.names = FALSE)
>  write.csv(mig2, paste(name.mig2, ".csv", sep = ""), row.names =
FALSE)
>  write.csv(Lmig, paste(name.Lmig, ".csv", sep = ""), row.names =
FALSE)
>  write.csv(Prop.types, paste(name.Prop.types, ".csv", sep = ""),
row.names = FALSE)
>  assign(name.st, st)
>  assign(name.wob, wob)
>  assign(name.mig, mig)
>  assign(name.mig2, mig2)
>  assign(name.Lmig, Lmig)
>  assign(name.Prop.types, Prop.types)
>}
>
>
># Group all samples together into one file for each statistic of
interest
>## Create a list of data frames containing all statistics of interest
for all samples
>files.all <- list.files(wdir, pattern = "*Allstats.merged.csv",
full.names = FALSE) # Get all Allstats.merged files
>files.all
>ldf.all <- sapply(files.all, read.csv, simplify = FALSE, USE.NAMES =
TRUE) # Read the files and store output in a list
>ldf.all <- lapply(ldf.all, as.data.frame, col.names = c("ID",
"Track.Length", "Track.Speed.Mean", "Track.Straightness", "MaxDis")) #
Ensure each element of the list is a data frame and assign clear column
names
>ldf.all # Check output
>
>
>## Create a data frame containing the Length statistic for all samples
and all cells.
>### As all films do not contain the same number of cells (tracks) we
need to create a base data frame where the first column is very long
>### We use the Length statistic of the first sample as the base
>all.Length <- data.frame(ldf.all[[1]]$Track.Length)
>head(all.Length)
>colnames(all.Length)[1] <- gsub("_Allstats.merged.csv", "",
names(ldf.all[1])) # Rename the column with just the sample's name
>dim(all.Length)
>head(all.Length)
>
>### Expand the data frame (just one column for now) with NA values
long enough that it can be merged with very long columns
>NA.vec <- rep(NA, 10000) # Creates a vector with 10 000 "NA" values
(10000 is long enough unless one or more of your analysed samples
include more than 10000 track)
>NA.df <- as.data.frame(NA.vec) # Transform the vector to a data frame
```

```
> (one column)
>colnames(NA.df)[1] <- colnames(all.Length)[1] # Set the NA data frame column name
>dim(NA.df)
>all.Length <- bind_rows(all.Length, NA.df) # Bind the two data frames so that the NA values are now below the stat values
>all.Length
>dim(all.Length) # Check output
>
>### Add a new columns for the Length statistic of each other sample.
>for (i in 2:length(ldf.all)){ # This is iterated from file [[2]] (second sample) since file [[1]] (first sample) was used to create the basis of the dataframe
>  all.Length$sample.name <- c(ldf.all[[i]]$Track.Length, rep(NA, nrow(all.Length) - length(ldf.all[[i]]$Track.Length))) # The newly added column contain the stat value and a number of NA value equal tho the length of the column - the number of stat values
>  colnames(all.Length)[i] <- gsub("_Allstats.merged.csv", "", names(ldf.all[i])) # Set the new  column name to the sample's name
>}
>
>head(all.Length) # Check output
>
>## Create a data frame containing the Mean Speed statistic for all samples and all cells.
>all.Speed.Mean <- data.frame(ldf.all[[1]]$Track.Speed.Mean)
>head(all.Speed.Mean)
>colnames(all.Speed.Mean)[1] <- gsub("_Allstats.merged.csv", "", names(ldf.all[1]))
>dim(all.Speed.Mean)
>head(all.Speed.Mean)
>NA.vec <- rep(NA, 10000)
>NA.df <- as.data.frame(NA.vec)
>colnames(NA.df)[1] <- colnames(all.Speed.Mean)[1]
>dim(NA.df)
>all.Speed.Mean <- bind_rows(all.Speed.Mean, NA.df)
>dim(all.Speed.Mean)
>head(all.Speed.Mean)
>
>length(all.Speed.Mean$`1038 NM`)
>head(all.Speed.Mean)
>
>for (i in 2:length(ldf.all)){
>  all.Speed.Mean$sample.name <- c(ldf.all[[i]]$Track.Speed.Mean, rep(NA, nrow(all.Speed.Mean) - length(ldf.all[[i]]$Track.Speed.Mean)))
>  colnames(all.Speed.Mean)[i] <- gsub("_Allstats.merged.csv", "", names(ldf.all[i]))
>}
>
>head(all.Speed.Mean)
>
>## Create a data frame containing the Straightness statistic for all samples and all cells.
```

```
>all.Straightness <- data.frame(ldf.all[[1]]$Track.Straightness)
>head(all.Straightness)
>colnames(all.Straightness)[1] <- gsub("_Allstats.merged.csv", "",
names(ldf.all[1]))
>dim(all.Straightness)
>head(all.Straightness)
>NA.vec <- rep(NA, 10000)
>NA.df <- as.data.frame(NA.vec)
>colnames(NA.df)[1] <- colnames(all.Straightness)[1]
>dim(NA.df)
>all.Straightness <- bind_rows(all.Straightness, NA.df)
>dim(all.Straightness)
>head(all.Straightness)
>
>length(all.Straightness$`1038 NM`)
>head(all.Straightness)
>
>for (i in 2:length(ldf.all)){
>  all.Straightness$sample.name <- c(ldf.all[[i]]$Track.Straightness,
rep(NA, nrow(all.Straightness) -
length(ldf.all[[i]]$Track.Straightness)))
>  colnames(all.Straightness)[i] <- gsub("_Allstats.merged.csv", "",
names(ldf.all[i]))
>}
>
>head(all.Straightness)
>
>
>## Create a data frame containing the Length statistic for only static
cells for all samples.
>files.st <- list.files(wdir, pattern = "*st.csv", full.names = FALSE)
>files.st
>ldf.st <- sapply(files.st, read.csv, simplify = FALSE, USE.NAMES =
TRUE)
>ldf.st <- lapply(ldf.st, as.data.frame, col.names = c("ID",
"Track.Length", "Track.Speed.Mean", "Track.Straightness",
"Track.Displacement.Length"))
>ldf.st
>as.list(ldf.st)
>
>st.Length <- data.frame(ldf.st[[1]]$Track.Length)
>head(st.Length)
>colnames(st.Length)[1] <- gsub("_st.csv", "", names(ldf.st[1]))
>dim(st.Length)
>head(st.Length)
>NA.vec <- rep(NA, 10000)
>NA.df <- as.data.frame(NA.vec)
>colnames(NA.df)[1] <- colnames(st.Length)[1]
>dim(NA.df)
>st.Length <- bind_rows(st.Length, NA.df)
>dim(st.Length)
>head(st.Length)
>
```

```
>length(st.Length$`1038 NM`)
>head(st.Length)
>
>for (i in 2:length(ldf.st)){
>  st.Length$sample.name <- c(ldf.st[[i]]$Track.Length, rep(NA,
nrow(st.Length) - length(ldf.st[[i]]$Track.Length)))
>  colnames(st.Length)[i] <- gsub("_st.csv", "", names(ldf.st[i]))
>}
>
>head(st.Length)
>
>## Create a data frame containing the Mean Speed statistic for only
static cells for all samples.
>st.Speed.Mean <- data.frame(ldf.st[[1]]$Track.Speed.Mean)
>head(st.Speed.Mean)
>colnames(st.Speed.Mean)[1] <- gsub("_st.csv", "", names(ldf.st[1]))
>dim(st.Speed.Mean)
>head(st.Speed.Mean)
>NA.vec <- rep(NA, 10000)
>NA.df <- as.data.frame(NA.vec)
>colnames(NA.df)[1] <- colnames(st.Speed.Mean)[1]
>dim(NA.df)
>st.Speed.Mean <- bind_rows(st.Speed.Mean, NA.df)
>dim(st.Speed.Mean)
>head(st.Speed.Mean)
>
>
>for (i in 2:length(ldf.st)){
>  st.Speed.Mean$sample.name <- c(ldf.st[[i]]$Track.Speed.Mean, rep(NA,
nrow(st.Speed.Mean) - length(ldf.st[[i]]$Track.Speed.Mean)))
>  colnames(st.Speed.Mean)[i] <- gsub("_st.csv", "", names(ldf.st[i]))
>}
>
>head(st.Speed.Mean)
>
>## Create a data frame containing the Straightness statistic for only
static cells for all samples.
>st.Straightness <- data.frame(ldf.st[[1]]$Track.Straightness)
>head(st.Straightness)
>colnames(st.Straightness)[1] <- gsub("_st.csv", "", names(ldf.st[1]))
>dim(st.Straightness)
>head(st.Straightness)
>NA.vec <- rep(NA, 10000)
>NA.df <- as.data.frame(NA.vec)
>colnames(NA.df)[1] <- colnames(st.Straightness)[1]
>dim(NA.df)
>st.Straightness <- bind_rows(st.Straightness, NA.df)
>dim(st.Straightness)
>head(st.Straightness)
>
>length(st.Straightness$`1038 NM`)
>head(st.Straightness)
>
```

```
>for (i in 2:length(ldf.st)){
>  st.Straightness$sample.name <- c(ldf.st[[i]]$Track.Straightness,
rep(NA, nrow(st.Straightness) -
length(ldf.st[[i]]$Track.Straightness)))
>  colnames(st.Straightness)[i] <- gsub("_st.csv", "",
names(ldf.st[i]))
>}
>
>head(st.Straightness)
>
>
>## Create a data frame containing the Length statistic for only
wobbling cells for all samples.
>files.wob <- list.files(wdir, pattern = "*wob.csv", full.names =
FALSE)
>files.wob
>ldf.wob <- sapply(files.wob, read.csv, simplify = FALSE, USE.NAMES =
TRUE)
>ldf.wob <- lapply(ldf.wob, as.data.frame, col.names = c("ID",
"Track.Length", "Track.Speed.Mean", "Track.Straightness",
"Track.Displacement.Length"))
>ldf.wob
>as.list(ldf.wob)
>
>wob.Length <- data.frame(ldf.wob[[1]]$Track.Length)
>head(wob.Length)
>colnames(wob.Length)[1] <- gsub("_wob.csv", "", names(ldf.wob[1]))
>dim(wob.Length)
>head(wob.Length)
>NA.vec <- rep(NA, 10000)
>NA.df <- as.data.frame(NA.vec)
>colnames(NA.df)[1] <- colnames(wob.Length)[1]
>dim(NA.df)
>wob.Length <- bind_rows(wob.Length, NA.df)
>dim(wob.Length)
>head(wob.Length)
>
>length(wob.Length$`1038 NM`)
>head(wob.Length)
>
>for (i in 2:length(ldf.wob)){
>  wob.Length$sample.name <- c(ldf.wob[[i]]$Track.Length, rep(NA,
nrow(wob.Length) - length(ldf.wob[[i]]$Track.Length)))
>  colnames(wob.Length)[i] <- gsub("_wob.csv", "", names(ldf.wob[i]))
>}
>
>head(wob.Length)
>
>## Create a data frame containing the Mean Speed statistic for only
wobbling cells for all samples.
>wob.Speed.Mean <- data.frame(ldf.wob[[1]]$Track.Speed.Mean)
>head(wob.Speed.Mean)
>colnames(wob.Speed.Mean)[1] <- gsub("_wob.csv", "", names(ldf.wob[1]))
>dim(wob.Speed.Mean)
```

```
>NA.vec <- rep(NA, 10000)
>NA.df <- as.data.frame(NA.vec)
>colnames(NA.df)[1] <- colnames(wob.Speed.Mean)[1]
>dim(NA.df)
>wob.Speed.Mean <- bind_rows(wob.Speed.Mean, NA.df)
>dim(wob.Speed.Mean)
>head(wob.Speed.Mean)
>
>length(wob.Speed.Mean$`1038 NM`)
>head(wob.Speed.Mean)
>
>for (i in 2:length(ldf.wob)){
>  wob.Speed.Mean$sample.name <- c(ldf.wob[[i]]$Track.Speed.Mean,
rep(NA, nrow(wob.Speed.Mean) - length(ldf.wob[[i]]$Track.Speed.Mean)))
>  colnames(wob.Speed.Mean)[i] <- gsub("_wob.csv", "",
names(ldf.wob[i]))
>}
>
>head(wob.Speed.Mean)
>
>## Create a data frame containing the Straightness statistic for only
wobbling cells for all samples.
>wob.Straightness <- data.frame(ldf.wob[[1]]$Track.Straightness)
>head(wob.Straightness)
>colnames(wob.Straightness)[1] <- gsub("_wob.csv", "",
names(ldf.wob[1]))
>dim(wob.Straightness)
>head(wob.Straightness)
>NA.vec <- rep(NA, 10000)
>NA.df <- as.data.frame(NA.vec)
>colnames(NA.df)[1] <- colnames(wob.Straightness)[1]
>dim(NA.df)
>wob.Straightness <- bind_rows(wob.Straightness, NA.df)
>dim(wob.Straightness)
>head(wob.Straightness)
>
>length(wob.Straightness$`1038 NM`)
>head(wob.Straightness)
>
>for (i in 2:length(ldf.wob)){
>  wob.Straightness$sample.name <- c(ldf.wob[[i]]$Track.Straightness,
rep(NA, nrow(wob.Straightness) -
length(ldf.wob[[i]]$Track.Straightness)))
>  colnames(wob.Straightness)[i] <- gsub("_wob.csv", "",
names(ldf.wob[i]))
>}
>
>head(wob.Straightness)
>
>
>## Create a data frame containing the Length statistic for only
migrating cells for all samples.
>files.mig <- list.files(wdir, pattern = "*mig.csv", full.names =
FALSE)
```

```
>files.mig
>ldf.mig <- sapply(files.mig, read.csv, simplify = FALSE, USE.NAMES =
TRUE)
>ldf.mig <- lapply(ldf.mig, as.data.frame, col.names = c("ID",
"Track.Length", "Track.Speed.Mean", "Track.Straightness",
"Track.Displacement.Length"))
>ldf.mig
>as.list(ldf.mig)
>
>mig.Length <- data.frame(ldf.mig[[1]]$Track.Length)
>head(mig.Length)
>colnames(mig.Length)[1] <- gsub("_mig.csv", "", names(ldf.mig[1]))
>dim(mig.Length)
>head(mig.Length)
>NA.vec <- rep(NA, 10000)
>NA.df <- as.data.frame(NA.vec)
>colnames(NA.df)[1] <- colnames(mig.Length)[1]
>dim(NA.df)
>mig.Length <- bind_rows(mig.Length, NA.df)
>dim(mig.Length)
>head(mig.Length)
>
>length(mig.Length$`1038 NM`)
>head(mig.Length)
>
>for (i in 2:length(ldf.mig)){
>  mig.Length$sample.name <- c(ldf.mig[[i]]$Track.Length, rep(NA,
nrow(mig.Length) - length(ldf.mig[[i]]$Track.Length)))
>  colnames(mig.Length)[i] <- gsub("_mig.csv", "", names(ldf.mig[i]))
>}
>
>head(mig.Length)
>
>## Create a data frame containing the Mean Speed statistic for only
migrating cells for all samples.
>mig.Speed.Mean <- data.frame(ldf.mig[[1]]$Track.Speed.Mean)
>head(mig.Speed.Mean)
>colnames(mig.Speed.Mean)[1] <- gsub("_mig.csv", "", names(ldf.mig[1]))
>dim(mig.Speed.Mean)
>head(mig.Speed.Mean)
>NA.vec <- rep(NA, 10000)
>NA.df <- as.data.frame(NA.vec)
>colnames(NA.df)[1] <- colnames(mig.Speed.Mean)[1]
>dim(NA.df)
>mig.Speed.Mean <- bind_rows(mig.Speed.Mean, NA.df)
>dim(mig.Speed.Mean)
>head(mig.Speed.Mean)
>
>length(mig.Speed.Mean$`1038 NM`)
>head(mig.Speed.Mean)
>
>for (i in 2:length(ldf.mig)){
>  mig.Speed.Mean$sample.name <- c(ldf.mig[[i]]$Track.Speed.Mean,
```

```
>rep(NA, nrow(mig.Speed.Mean) - length(ldf.mig[[i]]$Track.Speed.Mean)))
>  colnames(mig.Speed.Mean)[i] <- gsub("_mig.csv", "",
names(ldf.mig[i]))
>}
>
>head(mig.Speed.Mean)
>
>## Create a data frame containing the Straightness statistic for only
migrating cells for all samples.
>mig.Straightness <- data.frame(ldf.mig[[1]]$Track.Straightness)
>head(mig.Straightness)
>colnames(mig.Straightness)[1] <- gsub("_mig.csv", "",
names(ldf.mig[1]))
>dim(mig.Straightness)
>head(mig.Straightness)
>NA.vec <- rep(NA, 10000)
>NA.df <- as.data.frame(NA.vec)
>colnames(NA.df)[1] <- colnames(mig.Straightness)[1]
>dim(NA.df)
>mig.Straightness <- bind_rows(mig.Straightness, NA.df)
>dim(mig.Straightness)
>head(mig.Straightness)
>
>length(mig.Straightness$`1038 NM`)
>head(mig.Straightness)
>
>for (i in 2:length(ldf.mig)){
>  mig.Straightness$sample.name <- c(ldf.mig[[i]]$Track.Straightness,
rep(NA, nrow(mig.Straightness) -
length(ldf.mig[[i]]$Track.Straightness)))
>  colnames(mig.Straightness)[i] <- gsub("_mig.csv", "",
names(ldf.mig[i]))
>}
>
>head(mig.Straightness)
>
>
>## Create a data frame containing the Length statistic for only
migrating + Long migrating cells for all samples.
>files.mig2 <- list.files(wdir, pattern = "*mig2.csv", full.names =
FALSE)
>files.mig2
>ldf.mig2 <- sapply(files.mig2, read.csv, simplify = FALSE, USE.NAMES =
TRUE)
>ldf.mig2 <- lapply(ldf.mig2, as.data.frame, col.names = c("ID",
"Track.Length", "Track.Speed.Mean", "Track.Straightness",
"Track.Displacement.Length"))
>ldf.mig2
>as.list(ldf.mig2)
>
>mig2.Length <- data.frame(ldf.mig2[[1]]$Track.Length)
>head(mig2.Length)
>colnames(mig2.Length)[1] <- gsub("_mig2.csv", "", names(ldf.mig2[1]))
```

```
>dim(mig2.Length)
>head(mig2.Length)
>NA.vec <- rep(NA, 10000)
>NA.df <- as.data.frame(NA.vec)
>colnames(NA.df)[1] <- colnames(mig2.Length)[1]
>dim(NA.df)
>mig2.Length <- bind_rows(mig2.Length, NA.df)
>dim(mig2.Length)
>head(mig2.Length)
>
>length(mig2.Length$`1038 NM`)
>head(mig2.Length)
>
>for (i in 2:length(ldf.mig2)){
>  mig2.Length$sample.name <- c(ldf.mig2[[i]]$Track.Length, rep(NA,
nrow(mig2.Length) - length(ldf.mig2[[i]]$Track.Length)))
>  colnames(mig2.Length)[i] <- gsub("_mig2.csv", "",
names(ldf.mig2[i]))
>}
>
>head(mig2.Length)
>
>### Create a data frame containing the Mean Speed statistic for only
migrating + Long migrating cells for all samples.
>mig2.Speed.Mean <- data.frame(ldf.mig2[[1]]$Track.Speed.Mean)
>head(mig2.Speed.Mean)
>colnames(mig2.Speed.Mean)[1] <- gsub("_mig2.csv", "",
names(ldf.mig2[1]))
>dim(mig2.Speed.Mean)
>head(mig2.Speed.Mean)
>NA.vec <- rep(NA, 10000)
>NA.df <- as.data.frame(NA.vec)
>colnames(NA.df)[1] <- colnames(mig2.Speed.Mean)[1]
>dim(NA.df)
>mig2.Speed.Mean <- bind_rows(mig2.Speed.Mean, NA.df)
>dim(mig2.Speed.Mean)
>head(mig2.Speed.Mean)
>
>length(mig2.Speed.Mean$`1038 NM`)
>head(mig2.Speed.Mean)
>
>for (i in 2:length(ldf.mig2)){
>  mig2.Speed.Mean$sample.name <- c(ldf.mig2[[i]]$Track.Speed.Mean,
rep(NA, nrow(mig2.Speed.Mean) -
length(ldf.mig2[[i]]$Track.Speed.Mean)))
>  colnames(mig2.Speed.Mean)[i] <- gsub("_mig2.csv", "",
names(ldf.mig2[i]))
>}
>
>head(mig2.Speed.Mean)
>
>## Create a data frame containing the Straightness statistic for only
migrating + Long migrating cells for all samples.
>mig2.Straightness <- data.frame(ldf.mig2[[1]]$Track.Straightness)
```

```
>head(mig2.Straightness)
>colnames(mig2.Straightness)[1] <- gsub("_mig2.csv", "",
names(ldf.mig2[1]))
>dim(mig2.Straightness)
>head(mig2.Straightness)
>NA.vec <- rep(NA, 10000)
>NA.df <- as.data.frame(NA.vec)
>colnames(NA.df)[1] <- colnames(mig2.Straightness)[1]
>dim(NA.df)
>mig2.Straightness <- bind_rows(mig2.Straightness, NA.df)
>dim(mig2.Straightness)
>head(mig2.Straightness)
>
>length(mig2.Straightness$`1038 NM`)
>head(mig2.Straightness)
>
>for (i in 2:length(ldf.mig2)){
>  mig2.Straightness$sample.name <- c(ldf.mig2[[i]]$Track.Straightness,
rep(NA, nrow(mig2.Straightness) -
length(ldf.mig2[[i]]$Track.Straightness)))
>  colnames(mig2.Straightness)[i] <- gsub("_mig2.csv", "",
names(ldf.mig2[i]))
>}
>
>head(mig2.Straightness)
>
>
>## Create a data frame containing the Length statistic for only Long
migrating cells for all samples.
>files.Lmig <- list.files(wdir, pattern = "*Lmig.csv", full.names =
FALSE)
>files.Lmig
>ldf.Lmig <- sapply(files.Lmig, read.csv, simplify = FALSE, USE.NAMES =
TRUE)
>ldf.Lmig <- lapply(ldf.Lmig, as.data.frame, col.names = c("ID",
"Track.Length", "Track.Speed.Mean", "Track.Straightness",
"Track.Displacement.Length"))
>ldf.Lmig
>as.list(ldf.Lmig)
>
>Lmig.Length <- data.frame(ldf.Lmig[[1]]$Track.Length)
>head(Lmig.Length)
>colnames(Lmig.Length)[1] <- gsub("_Lmig.csv", "", names(ldf.Lmig[1]))
>dim(Lmig.Length)
>head(Lmig.Length)
>NA.vec <- rep(NA, 10000)
>NA.df <- as.data.frame(NA.vec)
>colnames(NA.df)[1] <- colnames(Lmig.Length)[1]
>dim(NA.df)
>Lmig.Length <- bind_rows(Lmig.Length, NA.df)
>dim(Lmig.Length)
>head(Lmig.Length)
>
```

```
>) - length(ldf.mig2[[i]]$Track.Straightness)))
>  colnames(mig2.Straightness)[i] <- gsub("_mig2.csv", "",
names(ldf.mig2[i]))
>}
>
>head(mig2.Straightness)
>
>
>## Create a data frame containing the Length statistic for only Long
migrating cells for all samples.
>files.Lmig <- list.files(wdir, pattern = "*Lmig.csv", full.names =
FALSE)
>files.Lmig
>ldf.Lmig <- sapply(files.Lmig, read.csv, simplify = FALSE, USE.NAMES =
TRUE)
>ldf.Lmig <- lapply(ldf.Lmig, as.data.frame, col.names = c("ID",
"Track.Length", "Track.Speed.Mean", "Track.Straightness",
"Track.Displacement.Length"))
>ldf.Lmig
>as.list(ldf.Lmig)
>
>Lmig.Length <- data.frame(ldf.Lmig[[1]]$Track.Length)
>head(Lmig.Length)
>colnames(Lmig.Length)[1] <- gsub("_Lmig.csv", "", names(ldf.Lmig[1]))
>dim(Lmig.Length)
>head(Lmig.Length)
>NA.vec <- rep(NA, 10000)
>NA.df <- as.data.frame(NA.vec)
>colnames(NA.df)[1] <- colnames(Lmig.Length)[1]
>dim(NA.df)
>Lmig.Length <- bind_rows(Lmig.Length, NA.df)
>dim(Lmig.Length)
>head(Lmig.Length)
>
>length(Lmig.Length$`1038 NM`)
>head(Lmig.Length)
>
>for (i in 2:length(ldf.Lmig)){
>  Lmig.Length$sample.name <- c(ldf.Lmig[[i]]$Track.Length, rep(NA,
nrow(Lmig.Length) - length(ldf.Lmig[[i]]$Track.Length)))
>  colnames(Lmig.Length)[i] <- gsub("_Lmig.csv", "",
names(ldf.Lmig[i]))
>}
>
>head(Lmig.Length)
>
>## Create a data frame containing the Mean Speed statistic for only
Long migrating cells for all samples.
>Lmig.Speed.Mean <- data.frame(ldf.Lmig[[1]]$Track.Speed.Mean)
>head(Lmig.Speed.Mean)
>colnames(Lmig.Speed.Mean)[1] <- gsub("_Lmig.csv", "",
names(ldf.Lmig[1]))
>dim(Lmig.Speed.Mean)
>head(Lmig.Speed.Mean)
```

```
>NA.vec <- rep(NA, 10000)
>NA.df <- as.data.frame(NA.vec)
>colnames(NA.df)[1] <- colnames(Lmig.Speed.Mean)[1]
>dim(NA.df)
>Lmig.Speed.Mean <- bind_rows(Lmig.Speed.Mean, NA.df)
>dim(Lmig.Speed.Mean)
>head(Lmig.Speed.Mean)
>
>length(Lmig.Speed.Mean$`1038 NM`)
>head(Lmig.Speed.Mean)
>
>for (i in 2:length(ldf.Lmig)){
>  Lmig.Speed.Mean$sample.name <- c(ldf.Lmig[[i]]$Track.Speed.Mean,
rep(NA, nrow(Lmig.Speed.Mean) -
length(ldf.Lmig[[i]]$Track.Speed.Mean)))
>  colnames(Lmig.Speed.Mean)[i] <- gsub("_Lmig.csv", "",
names(ldf.Lmig[i]))
>}
>
>head(Lmig.Speed.Mean)
>
>## Create a data frame containing the Straightness statistic for only
Long migrating cells for all samples.
>Lmig.Straightness <- data.frame(ldf.Lmig[[1]]$Track.Straightness)
>head(Lmig.Straightness)
>colnames(Lmig.Straightness)[1] <- gsub("_Lmig.csv", "",
names(ldf.Lmig[1]))
>dim(Lmig.Straightness)
>head(Lmig.Straightness)
>NA.vec <- rep(NA, 10000)
>NA.df <- as.data.frame(NA.vec)
>colnames(NA.df)[1] <- colnames(Lmig.Straightness)[1]
>dim(NA.df)
>Lmig.Straightness <- bind_rows(Lmig.Straightness, NA.df)
>dim(Lmig.Straightness)
>head(Lmig.Straightness)
>
>length(Lmig.Straightness$`1038 NM`)
>head(Lmig.Straightness)
>
>for (i in 2:length(ldf.Lmig)){
>  Lmig.Straightness$sample.name <- c(ldf.Lmig[[i]]$Track.Straightness,
rep(NA, nrow(Lmig.Straightness) -
length(ldf.Lmig[[i]]$Track.Straightness)))
>  colnames(Lmig.Straightness)[i] <- gsub("_Lmig.csv", "",
names(ldf.Lmig[i]))
>}
>
>head(Lmig.Straightness)
>
>
># Write all outputs as .csv files
>write.csv(all.Length, "all.Length.csv", row.names = FALSE)
```

```
>write.csv(all.Speed.Mean, "all.Speed.Mean.csv", row.names = FALSE)
>write.csv(all.Straightness, "all.Straightness.csv", row.names = FALSE)
>write.csv(st.Length, "st.Length.csv", row.names = FALSE)
>write.csv(st.Speed.Mean, "st.Speed.Mean.csv", row.names = FALSE)
>write.csv(st.Straightness, "st.Straightness.csv", row.names = FALSE)
>write.csv(wob.Length, "wob.Length.csv", row.names = FALSE)
>write.csv(wob.Speed.Mean, "wob.Speed.Mean.csv", row.names = FALSE)
>write.csv(wob.Straightness, "wob.Straightness.csv", row.names = FALSE)
>write.csv(mig.Length, "mig.Length.csv", row.names = FALSE)
>write.csv(mig.Speed.Mean, "mig.Speed.Mean.csv", row.names = FALSE)
>write.csv(mig.Straightness, "mig.Straightness.csv", row.names = FALSE)
>write.csv(mig2.Length, "mig2.Length.csv", row.names = FALSE)
>write.csv(mig2.Speed.Mean, "mig2.Speed.Mean.csv", row.names = FALSE)
>write.csv(mig2.Straightness, "mig2.Straightness.csv", row.names =
FALSE)
>write.csv(Lmig.Length, "Lmig.Length.csv", row.names = FALSE)
>write.csv(Lmig.Speed.Mean, "Lmig.Speed.Mean.csv", row.names = FALSE)
>write.csv(Lmig.Straightness, "Lmig.Straightness.csv", row.names =
FALSE)
>
># Organize data to have one data point per sample (the mean) for all
cells
>all.Length.mean <- as.data.frame(colMeans(all.Length, na.rm = TRUE)) #
Creates a data frame with the mean value for each samples
>all.Length.mean
>
>all.Speed.Mean.mean <- as.data.frame(colMeans(all.Speed.Mean, na.rm =
TRUE)) # Creates a data frame with the mean value for each samples
>all.Speed.Mean.mean
>
>all.Straightness.mean <- as.data.frame(colMeans(all.Straightness,
na.rm = TRUE)) # Creates a data frame with the mean value for each
samples
>all.Straightness.mean
>
># Organize data to have one data point per sample (the mean) for
static cells
>st.Length.mean <- as.data.frame(colMeans(st.Length, na.rm = TRUE)) #
Creates a data frame with the mean value for each samples
>st.Length.mean
>
>st.Speed.Mean.mean <- as.data.frame(colMeans(st.Speed.Mean, na.rm =
TRUE)) # Creates a data frame with the mean value for each samples
>st.Speed.Mean.mean
>
>st.Straightness.mean <- as.data.frame(colMeans(st.Straightness, na.rm
= TRUE)) # Creates a data frame with the mean value for each samples
>st.Straightness.mean
>
># Organize data to have one data point per sample (the mean) for
wobbling cells
>wob.Length.mean <- as.data.frame(colMeans(wob.Length, na.rm = TRUE)) #
Creates a data frame with the mean value for each samples
>wob.Length.mean
```

```
>wob.Speed.Mean.mean <- as.data.frame(colMeans(wob.Speed.Mean, na.rm =
TRUE)) # Creates a data frame with the mean value for each samples
>wob.Speed.Mean.mean
>
>wob.Straightness.mean <- as.data.frame(colMeans(wob.Straightness,
na.rm = TRUE)) # Creates a data frame with the mean value for each
samples
>wob.Straightness.mean
>
># Organize data to have one data point per sample (the mean) for
migrating cells
>mig.Length.mean <- as.data.frame(colMeans(mig.Length, na.rm = TRUE)) #
Creates a data frame with the mean value for each samples
>mig.Length.mean
>
>mig.Speed.Mean.mean <- as.data.frame(colMeans(mig.Speed.Mean, na.rm =
TRUE)) # Creates a data frame with the mean value for each samples
>mig.Speed.Mean.mean
>
>mig.Straightness.mean <- as.data.frame(colMeans(mig.Straightness,
na.rm = TRUE)) # Creates a data frame with the mean value for each
samples
>mig.Straightness.mean
>
># Organize data to have one data point per sample (the mean) for
migrating + Long migrating cells
>mig2.Length.mean <- as.data.frame(colMeans(mig2.Length, na.rm = TRUE))
# Creates a data frame with the mean value for each samples
>mig2.Length.mean
>
>mig2.Speed.Mean.mean <- as.data.frame(colMeans(mig2.Speed.Mean, na.rm
= TRUE)) # Creates a data frame with the mean value for each samples
>mig2.Speed.Mean.mean
>
>mig2.Straightness.mean <- as.data.frame(colMeans(mig2.Straightness,
na.rm = TRUE)) # Creates a data frame with the mean value for each
samples
>mig2.Straightness.mean
>
># Organize data to have one data point per sample (the mean) for Long
migrating cells
>Lmig.Length.mean <- as.data.frame(colMeans(Lmig.Length, na.rm = TRUE))
# Creates a data frame with the mean value for each samples
>Lmig.Length.mean
>
>Lmig.Speed.Mean.mean <- as.data.frame(colMeans(Lmig.Speed.Mean, na.rm
= TRUE)) # Creates a data frame with the mean value for each samples
>Lmig.Speed.Mean.mean
>
>Lmig.Straightness.mean <- as.data.frame(colMeans(Lmig.Straightness,
na.rm = TRUE)) # Creates a data frame with the mean value for each
samples
>Lmig.Straightness.mean
>
># Write outputs as .csv files
```

```
>write.csv(all.Length.mean, "all.Length.mean.csv", row.names = FALSE)
>write.csv(all.Speed.Mean.mean, "all.Speed.Mean.mean.csv", row.names =
FALSE)
>write.csv(all.Straightness.mean, "all.Straightness.mean.csv",
row.names = FALSE)
>write.csv(st.Length.mean, "st.Length.mean.csv", row.names = FALSE)
>write.csv(st.Speed.Mean.mean, "st.Speed.Mean.mean.csv", row.names =
FALSE)
>write.csv(st.Straightness.mean, "st.Straightness.mean.csv", row.names
= FALSE)
>write.csv(wob.Length.mean, "wob.Length.mean.csv", row.names = FALSE)
>write.csv(wob.Speed.Mean.mean, "wob.Speed.Mean.mean.csv", row.names =
FALSE)
>write.csv(wob.Straightness.mean, "wob.Straightness.mean.csv",
row.names = FALSE)
>write.csv(mig.Length.mean, "mig.Length.mean.csv", row.names = FALSE)
>write.csv(mig.Speed.Mean.mean, "mig.Speed.Mean.mean.csv", row.names =
FALSE)
>write.csv(mig.Straightness.mean, "mig.Straightness.mean.csv",
row.names = FALSE)
>write.csv(mig2.Length.mean, "mig2.Length.mean.csv", row.names = FALSE)
>write.csv(mig2.Speed.Mean.mean, "mig2.Speed.Mean.mean.csv", row.names
= FALSE)
>write.csv(mig2.Straightness.mean, "mig2.Straightness.mean.csv",
row.names = FALSE)
>write.csv(Lmig.Length.mean, "Lmig.Length.mean.csv", row.names = FALSE)
>write.csv(Lmig.Speed.Mean.mean, "Lmig.Speed.Mean.mean.csv", row.names
= FALSE)
>write.csv(Lmig.Straightness.mean, "Lmig.Straightness.mean.csv",
row.names = FALSE)
>
># Combine proportions files
>files.prop <- list.files(wdir, pattern = "*Proportions.csv",
full.names = FALSE) # Get proportion files
>files.prop
>ldf.prop <- sapply(files.prop, read.csv, simplify = FALSE, USE.NAMES =
TRUE) # Read proportion files
>ldf.prop
>Proportions <- do.call("rbind", ldf.prop) # Merge proportion files
together into one data frame
>Proportions # Check output
>
>write.csv(Proportions, "Proportions.csv", row.names = FALSE) # Write
output as a .csv file
```
